# The Therapeutic Principle of Combined Strengthening Qi and Eliminating Pathogens in Treating Middle-Advanced Primary Liver Cancer: A Systematic Review and Meta-Analysis

**DOI:** 10.3389/fphar.2021.714287

**Published:** 2021-10-27

**Authors:** Yingqi She, Qinfeng Huang, Zhen Ye, Yu Hu, Mingquan Wu, Kaihua Qin, Ailing Wei, Xin Yang, Yuyao Liu, Cuihan Zhang, Qiaobo Ye

**Affiliations:** ^1^ School of Basic Medical Sciences, Chengdu University of Traditional Chinese Medicine, Chengdu, China; ^2^ Department of Oncology, The First Affiliated Hospital, Guangxi University of Chinese Medicine, Nanning, China; ^3^ Department of Pharmacy, Sichuan Provincial Orthopedic Hospital, Chengdu, China; ^4^ Health Preservation and Rehabilitation College, Chengdu University of Traditional Chinese Medicine, Chengdu, China; ^5^ Department of Liver Disease, The First Affiliated Hospital, Guangxi University of Chinese Medicine, Nanning, China; ^6^ Pharmacy College, Chengdu University of Traditional Chinese Medicine, Chengdu, China

**Keywords:** Chinese medicinal formulas, transcatheter arterial chemoembolization, drastic medicinals, therapeutic principle, primary liver cancer, meta-analysis

## Abstract

**Background:** The combination of strengthening Qi and eliminating pathogens is an available therapeutic principle in traditional Chinese medicine (TCM) for primary liver cancer (PLC) at middle-advanced stage. However, there is a lack of reasonable evidence to support the proper application of this therapeutic principle. This meta-analysis aims to evaluate the efficacy and safety of Chinese medicinal formulas (CMFs), including two subgroup analyses of the principle of strengthening Qi and eliminating pathogens.

**Method:** Clinical trials were obtained through searching of EMBASE, Web of Science, PubMed, Cochrane Library, Chinese National Knowledge Infrastructure, Wanfang Database, Chinese Scientific Journal Database, Chinese Biomedical Literature Database, and two clinical trial registries. The randomized controlled trials with the combination of CMFs and transcatheter arterial chemoembolization (TACE) in the experiment group were acceptable, in contrast to the TACE alone in the control group. The statistics analysis was performed on Review Manager 5.4.

**Results:** A total of eligible 24 trials were accessed in this work. Overall, CMFs could improve the survival duration of 6 months, 1 year, and 2 years, Karnofsky Performance Status, tumor objective response rate (ORR), AFP, and symptom. In the subgroup analysis, trials complying with the principle of single strengthening Qi did not show any significant difference in increasing tumor ORR. Meanwhile, the principle of combined strengthening Qi and eliminating pathogens was uncertain in improving symptoms and 1-year and 2-year survival time. In addition, the outcome indexes of ALT and AST were heterogeneous. In last, the total occurrence of adverse events could not be reduced via using CMFs. Patients treated with CMFs exhibited liver injury, fever, and white blood cell decline, with mild events occurring more frequently and severe events occurring less.

**Conclusion:** CMFs are an effective treatment method to cure PLC at the middle-advanced stage. Adopting the principle of single strengthening Qi presents better efficacy in the long term by prolonging the survival duration. Following the principle of combined strengthening Qi and eliminating pathogens could be more beneficial to patients in short term by lessening the tumor size. CMFs have the advantage of reducing certain serious adverse events.

## Introduction

Primary liver cancer (PLC) is one of the common digestive system neoplasms, including most of the hepatocellular carcinoma (HCC), a small part of cholangiocarcinoma (CCC), and combined hepatocholangiocarcinoma (HCC-ICC). According to GLOBOCAN 2020 (Sung et al., 2021), 906,000 new PLC cases and 830,000 PLC death cases were reported. Based on cancer statistics published by the American Cancer Society in 2020 (Siegel et al., 2020), liver cancer (LC) was the fastest-growing one among tumor diseases, increasing at the rate of 2-3% per year during 2007–2016 in the United States. In all the regions and nations, China could not be ignored as it has the largest population of HCC patients (Zhu et al., 2016). Although both male and female morbidity and mortality dropped from 2000 to 2015 in China, the PLC still ranked fourth in new cases and second place in cancer mortality (Zhang et al., 2020).

Typically, due to the substantial compensation of hepatic function, PLC patients usually do not have clinical symptoms, such as liver pain and hepatomegaly, until the disease develops to the middle-late stage. Thus, a large number of patients miss the radical therapeutic time windows because of the delay in early diagnosis. With the innovation of medical technology, the lifespan of HCC patients has been extended (Tang et al., 2018); however, the burden of PLC should be paid more attention. From 2005 to 2015, absolute years of life lost in HCC increased 4.5% (Yang et al., 2019). Also, the countries with resource scarcity and those with a small number of doctors per 10,000 persons are more likely to face an early onset of LC (Are et al., 2017). For average families, the treatment of PLC can bring a huge economic burden. For countries, it also increases the financial burden on the public medical system and leads to the loss of social productivity.

Transcatheter Arterial Chemoembolization (TACE) has been regarded as the most common nonsurgical treatment for LC since Yamada was reported in Japan (Imai et al., 2014). And it has been the first-line treatment for PLC patients in the intermediate stage (BCLC stage B) (Galle et al., 2018). Although sorafenib is the current primary medicine for treating advanced hepatocellular carcinoma, BRIDGE’s real-world clinical research noted that almost 50% of patients, ranging from stage 0 to D, were treated with TACE (Raoul et al., 2019). As early as 2011, it was reported in the Asian Consensus Workshop that TACE and surgical resection have been applied to the treatment for advanced hepatocellular carcinoma in Korea, China, and Japan (Han et al., 2011). Therefore, there is no doubt that TACE is a standard treatment for middle-late stage PLC.

TACE is a treatment in which embolic agents and chemotherapy drugs are mixed together and injected from the hepatic artery to the tumor site, serving to embolize the tumor-feeding arteries and induce ischemic necrosis in the tumor tissue. Depending on the embolic agent, there are conventional TACE (cTACE) treatments using iodine oil and drug-eluting bead TACE (DEB-TACE) that enables drugs to sustainedly release up to a month (Raoul et al., 2019). Adriamycin, mitomycin, and cisplatin are three chemotherapeutic agents frequently used in TACE (Galle et al., 2017). It is common for patients to suffer from postembolization syndrome after TACE treatment because of the toxic effects from chemotherapeutic agents and the stress response caused by the embolization of local tissues. Approximately 35–100% of patients experience abdominal pain, fever, and nausea, and some of them choose to discontinue TACE therapy in response to severe adverse reactions and liver failure (Raoul et al., 2011). Mason also pointed out that the postembolization syndrome was associated with an increased risk of death (Mason et al., 2015). Meanwhile, the tumor hypoxic microenvironment, resulting from artery occlusion, can induce the release of angiogenic factors and promote tumor neovascularization (Bruix et al., 2015). Consequently, it is urgent to find new antihepatoma drugs or new therapies. Currently, the clinical application of combining traditional or complementary medicine with TACE therapy is widespread, especially in China. Nevertheless, the efficacy and safety of traditional Chinese medicine (TCM) still need evidence-based reviews to provide more informative clinical proof.

TCM is gaining popularity, especially in tumor patients, since TCM focuses on the whole body of the patient and aims to make the patient adapt to chemotherapy by improving the patient’s immunity and reducing side effects with the application of Chinese medicinal formulas (CMFs). Generally speaking, there are two common therapeutic principles for tumor patients, respectively, the principle of single strengthening Qi (PSSQ) and the principle of combined strengthening Qi and eliminating pathogens (PCSQEP). The former consists of Qi replenishing medicinals and the latter add drastic medicinals to Qi replenishing formulas to eliminate pathogens. Hitherto, there has been no evidence from evidence-based medicine indicating which treatment regimen is more beneficial for PLC patients at the intermediate-advanced stage when TCM is used in conjunction with TACE. In this meta-analysis, drastic medicinals were defined, including toxic medicinals recorded in the China Pharmacopeia and characterized by a strong effect on dispersing stagnant Qi, purging fluid, dissipating mass, and breaking blood stasis. Overall, there is a need to assess the efficacy and safety of CMFs and figure out the applicable conditions for PSSQ and PCSQEP.

## Methods

This study was conducted by the Preferred Reporting Items for Systematic Reviews and Meta-Analyses (PRISMA) statement (Moher et al., 2009).

### Search Strategies

The electronic search was from their inception until December 18, 2020, which was performed in the Chinese National Knowledge Infrastructure (CNKI), the Wanfang Database, the Chinese Scientific Journal Database (VIP), the Chinese Biomedical Literature Database (CBM), Excerpta Medica Database (EMBASE), Web of Science, PubMed, and Cochrane Library. The search formulation adopted a combination of free texts and medical subject heading (MeSH), which was developed to meet the requirements according to the search habits of each database. At the same time, there is no restriction of search formulation on language or country. The search formula for each database is shown in the Supplementary Material. References that meet the requirements would be taken into consideration as well. Two clinical trial registries (http://www.chictr.org.cn/and http://www.clinicaltrials.gov/) were also searched. We also contacted the trial registrants by email to try to obtain their raw data in case we missed some of the trials that were appropriate for this study.

### Study Selection

#### Types of Participants

All participants enrolled in the study met the diagnostic criteria for intermediate-advanced PLC, which meant that trials involving participants with secondary hepatic carcinoma or participants at an early stage were excluded. The studies that did not mention the staging of LC were also excluded.

#### Types of Intervention

All patients had to receive TACE treatment or symptomatic and supportive treatment after TACE. On the basis of the TACE, the experimental group was treated with CMFs containing Qi replenishing medicinals in basic formulas by oral, lasting at least 1 week. Studies were also excluded if the experimental group used single Chinese medicinal, acupuncture, moxibustion, qigong, plaster, Chinese medicine injections, psychological interventions, and other Western medicine. Trials that did not report prescriptions were also excluded.

#### Types of Comparison

Except for TACE treatment, other treatment plans in control groups such as TACE combined with surgical resection, radiofrequency ablation, radiotherapy, and targeted therapy were excluded.

#### Outcomes

Referring to the Guidelines for Clinical Research of Malignant Tumors on New Traditional Chinese Medicines (GCRMTNTCM), the 6-month, 1-year, and 2-year survival rates and the efficient rate in Karnofsky Performance Status (KPS) were the primary outcome. The secondary outcomes were the tumor objective response rate (ORR), ALT, AST, AFP, symptoms improvement, and adverse events. Adverse events included nausea and vomit, inappetence, digestive symptoms, fever, liver injury, white blood cell (WBC) reduction, platelet reduction, hemoglobin reduction, bone marrow suppression, and neurotoxicity. Trials without relevant outcomes and sufficient data for statistical analysis were excluded.

#### Type of Studies

Only randomized controlled trials (RCTs) were accepted. Animal experiments, meta-analyses, systematic reviews, retrospective studies, case reports, and reviews were not eligible.

#### Others

To improve the quality of each included trial and to protect efficacy from other factors, we also set the inclusive condition for clinical trials according to the approach of RCTs design (Supplementary Table S1). Only the clinical trials that meet the following requirements can be included: 1) all patients who received primary treatment or had undergone a washout period before trials entry; 2) studies with comparable baseline; 3) studies with diagnostic, inclusion, and exclusion criteria; 4) studies with efficacy evaluation standard. Additionally, the studies would be excluded, if they were derived from conference papers or dissertations.

### Data Collection and Extraction

First, She Y.Q. was responsible for searching according to the established search formulation, screening the eligible literature, and extracting the records. Second, Liu Y.Y. and Zhang C.H. independently screened the titles and abstracts of the trials and excluded those that were not compliant with the criteria. After merging the results of the two, She Y.Q. and Hu Y. proceeded to the full-text screening stage. When they encountered disagreement, it was handed over to the senior reviewers Wei A.L. for judgment. At the end of the screening process, all literature was reviewed or rechecked by the senior reviewers Qin K.H. Finally, from the included studies, information was extracted by two reviewers: Liu Y.Y. extracted the data, and She Y.Q. was in charge of reviewing it (see Supplementary Figure S1).

### Assessment of Risk of Bias

Regarding the Cochrane Handbook (Higgins et al., 2019), two reviewers independently analyzed the risk bias of the included trials. The following seven items were evaluated: 1) random sequence generation; 2) allocation concealment; 3) blinding of participants and personnel; 4) blinding of outcome assessment; 5) incomplete outcome data; 6) selective reporting; and 7) other biases. Each domain was graded on three levels based on methodological quality: high risk, low risk, and unclear risk.

### Statistical Analysis

RevMan5.4 and Stata 16 were the software used for meta-analysis. According to the outcome category, continuous data were calculated as a weighted mean difference (WMD) with a 95% confidence interval (CI), while categorical data were calculated as the risk ratio (RR) with 95% CI. *p* < 0.05 was considered statistically significant. Heterogeneity was assessed by the chi-square test and I^2^ statistic. When the *p* > 0.1 and I^2^ ≤ 50%, these outcomes were regarded as homogeneity, using a fixed effect model. Conversely, when the *p* ≤ 0.1 and I^2^ > 50%, these outcomes were deemed to have heterogeneity with a random effect model. A subgroup analysis was performed according to whether drastic medicinals were involved in the CMFs. Besides, if there were heterogeneity in the results, we would look for heterogeneity sources and perform a sensitivity analysis by converting the random effect model and fixed effect model to determine whether the results are stable. Publication bias was analyzed visually through funnel plots.

## Results

### Study Identification

**FIGURE 1:**
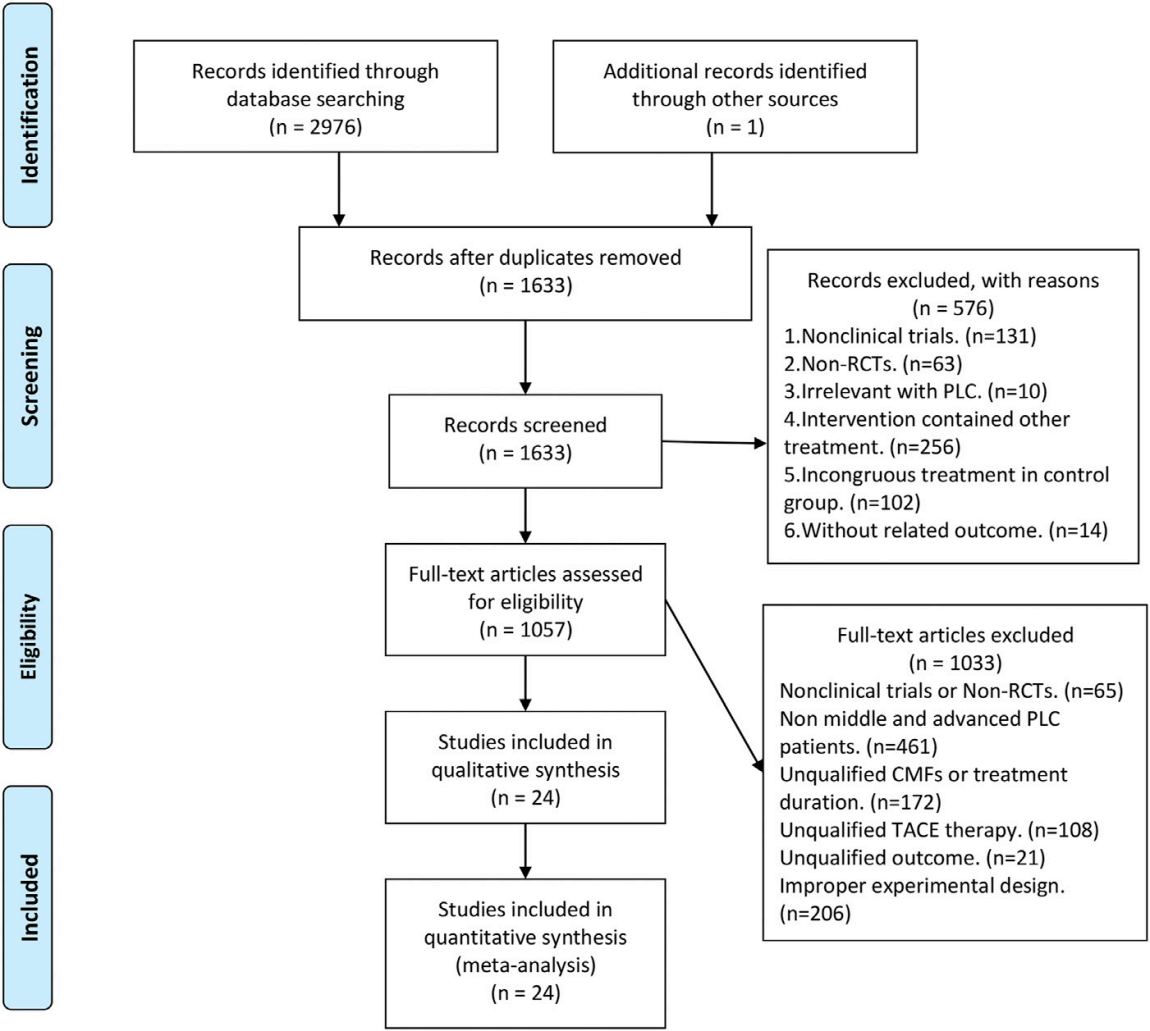
PRISMA flow diagram.

Figure 1 shows the flowchart of clinical trials screening in the meta-analysis, which was derived from the PRISMA statement (Moher et al., 2009). A total of 2,977 studies were obtained after searching in the database as well as hand retrieval, of which 1,344 were duplicates. 1,633 articles were entered into the records screening, and 576 articles were excluded. The remaining 1,057 articles were read in full text, where the more stringent inclusion criteria were implemented based on PICOS and experimental design (Supplementary Table S2), resulting in the exclusion of 1,033 articles. There were finally 24 eligible papers enrolled in this meta-analysis (Zhang et al., 2007; Huang et al., 2009; Chi et al., 2010; Li et al., 2011; Ding, 2012; Ji et al., 2012; Liu et al., 2013; Rong et al., 2013; Deng et al., 2014; Cheng, 2015; Feng and Chen, 2015; Tang et al., 2015; Wang, 2015; Ye et al., 2015; Wang et al., 2016; Li and Xu, 2017; Song et al., 2017; Zhang et al., 2017; Du et al., 2018; Jiang et al., 2020; Shen et al., 2020; Wang et al., 2020; Wang et al., 2020; Zhang, 2020).

### Study Characteristics

To conduct the research, the researchers enrolled 1,786 patients. The 24 included trials were all completed in China, with sample sizes ranging from 40 to 120. Baseline information for each trial was shown in Table 1, and clinical data were comparable across all studies. Neoplasm staging systems were mentioned in 21 among 24 trials, two studies used the criteria developed by the Union for International Cancer (UICC), and three studies utilized the Barcelona staging. The remaining 16 trials adopted the standard established in China. Although no staging criteria were reported, three further trials included PLC patients at the intermediate-advanced stage. Except for nine studies in which Child-Pugh scores were not recorded and one trial that enrolled patients with Child-Pugh class C, the residual studies were all Child-Pugh class A or B patients. The KPS recorded in all 19 articles was greater than 50, indicating that at least the patients in these studies were able to live on their own and did not require heavy dependence on others. The details of the intervention are shown in Table 2 and Supplementary Table S3. Regarding the choice of dosage form, two trials were administered in the form of capsules, one trial was administered in the form of oral solution, one trial was administered in the form of tablets, one trial was administered in the form of pills, and 19 trials were administered in the form of decoctions. As syndrome differentiation is one of the features of TCM, TCM physicians will modify the decoctions based on the severity of the disease and symptoms (e.g., abdominal distension, jaundice, and insomnia). As a result, 13 trials treated patients with the modified formulas, and six trials used the basic formulas. The proportion of included studies with and without drastic medicinals was 1:1. Furthermore, one trial did not account for the agents used for TACE. The duration of treatment ranged from 2 to 12 weeks. Patients either received TACE for the first time or experienced at least a 2-week washout period before enrolling in the trial.

**TABLE 1:** Basic characteristics of the included studies.

First author and year	Staging criteria	Stage	Sample size (E/C)	Age (year)	Male/female	Child-Pugh	KPS	Drastic medicinals added	Treatment period	Washout period	Outcome measure
Zhang (2020)	BCLC	CD	20/20	E:59.6 ± 9.3 C:59.6 ± 9.3	E:15/5 C:15/5	—	>70	No	8 w	4 w	abei
Shen L. N. (2020)	GDTPLCC (2017)	Ⅱb and Ⅲ	30/30	E:60.03 ± 9.48 C:58.37 ± 11.05	E:24/6 C:25/5	AB	—	No	5 w	Primary treatment	fgj
Feng and Chen (2015)	—	Ⅱ and Ⅲ	42/42	—	—	—	>60	No	12 w	Primary treatment	de
Cheng M. F. (2015)	DTCCCC (1991)	Ⅱ and Ⅲ	42/42	—	—	—	—	No	2 w	4 w	abfgj
Deng L. (2014)	CDSCPLCC (2001)	Ⅱ and Ⅲ	42/38	E:66.78 ± 3.98 C:65.68 ± 4.69	E:26/12 C:25/17	AB	≥60	No	12 w	4 w	abefghij
Liu X. (2013)	CPG·CS	Ⅱ and Ⅲ	32/32	E:51.7 ± 10.3 C:52.2 ± 9.74	E:27/5 C:26/6	AB	≥60	No	8 w	4 w	deij
Ji J. (2012)	DTCCCC (1991)	Ⅱ and Ⅲ	28/28	E:44.3 ± 11.6 C:43.6 ± 12.8	E:18/10 C:16/12	AB	≥60	No	4 w	8 w	dej
Li Y.H. (2011)	CDSCPLCC (2001)	Ⅱ and Ⅲ	38/36	E:52.7 C:54.1 (median age)	E:29/7 C:31/7	AB	≥60	No	60 d	4 w	abcdehij
Chi H. C. (2010)	DTCCCC (1991)	Middle-advanced	60/60	—	E:36/24 C:38/22	—	—	No	4 m	4 w	bcdej
Wang A. M. (2020)	BCLC	BC	30/30	E:49.24 ± 13.74 C:48.64 ± 14.48	C:18/12 E:17/13	AB	—	No	4 w	4 w	fgj
Song Y. N. (2017)	GDTPLCC (2017)	Middle-advanced	40/40	E:54.48 ± 8.36 C:53.7 ± 8.27	E:18/22 C:30/10	—	60–80	No	3 rounds, 6–8 w per round	Primary treatment	abcefgh
Ye W. D. (2015)	BCLC	BC	34/34	E:55.15 C:55.24 (median age)	E:29/5 C:30/4	—	≥70	No	5 w	4 w	efj
Huang J. Y. (2009)	DTCCCC (1990)	Ⅱ and Ⅲ	30/30	E:54.5 C:56.1	E:21/9 C:20/10	—	≥50	Yes	60 d	4 w	dej
Wang (2015)	GDTPLC (2011)	Ⅱ, Ⅲ, and Ⅳ	46/46	E:41.2 ± 11.7 C:40.6 ± 12.4	E:25/21 C:27/19	—	>50	Yes	4 w	2 w	eh
Rong Z. (2013)	NDDTCCCC	Middle-advanced	30/30	E:52.73 ± 10.42 C:55.37 ± 10.68	E:18/12 C:16/14	AB	≥60	Yes	8 w	4 w	efgh
Wang Q. M. (2016)	CPG·CS	Ⅲ and Ⅳ	30/30	E:46.67 ± 7.19 C:46.47 ± 7.38	E:24/6 C:22/8	AB	≥60	Yes	8 w	4 w	deij
Du H. P. (2018)	—	Ⅱ and Ⅲ	35/34	—	E:24/10 C:21/14	ABC	>60	Yes	3 m	Primary treatment	fgj
Wang X. D. (2020)	GDTPLCC (2017)	Ⅱb and Ⅲa	25/25	E:56.52 ± 13.40 C:55.52 ± 12.58	E:20/5C:23/2	AB	≥60	Yes	6 w	4 w	defghij
Jiang R. R. (2020)	TNM	Ⅱ and Ⅲ	45/45	E:50.6 ± 8.1 C:49.7 ± 8.0	E:25/20 C:23/22	AB	>75	Yes	12 w	Primary treatment or 4 w	ehj
Tang Q. Y. (2015)	CDSCPLCC (2002)	Middle-advanced	53/53	E:53.67 ± 11.18 C:55.38 ± 10.72	E:46/7 C:48/5	AB	≥60	Yes	12 w	8 w	defg
Ding R. F. (2012)	—	Ⅲ and Ⅳ	33/30	E:53.9 C:56.4	E:17/13 C:19/14	AB	—	Yes	2 rounds, 3–5 w per round	Primary treatment	e
Zhang Q. (2007)	DTCCCC (1991)	Middle-advanced	58/54	E:58.0 ± 7.0 C:56.5 ± 8.5	E:35/19 C:38/20	—	≥50	Yes	2 m	4 w	abcdej
Zhang Z. Y. (2017)	TNM	Ⅱ, Ⅲ, and Ⅳ	38/38	E:39.42 ± 5.37 C:39.61 ± 5.42	E:25/13 C:26/12	AB	>60	Yes	90 d	4 w	efgh
Li and Xu (2017)	GDTPLC (2011)	Middle-advanced	40/38	E:49.08 ± 11.27 C:46.18 ± 10.65	E:30/8 C:32/8	AB	≥60	Yes	60 d	Primary treatment	bcdj

BCLC, Barcelona clinic liver cancer; GDTPLCC (2017), Guidelines for Diagnosis and Treatment of Primary Liver Cancer in China (2017 Edition); DTCCCC (1991), the Diagnosis and Treatment Criterion for Common Cancer in China (1991 Edition); CDSCPLCC (2001), Clinical Diagnosis and Staging Criteria of Primary Liver Cancer in China (2001 Edition); CPG·CS, Clinical Practice Guideline·Cancer Section; DTCCCC (1990), the Diagnosis and Treatment Criterion for Common Cancer in China (1991 Edition); GDTPLC (2011), Guidelines on the Diagnosis and Treatment of Primary Liver Cancer (2011 Edition); NDDTCCCC, New edition of the Diagnosis and Treatment Criterion for Common Cancer in China (1999 Edition); TNM, TNM Classification of Malignant Tumors; CDSCPLCC (2002), Clinical Diagnosis and Staging Criteria of Primary Liver Cancer in China (2002 Edition); E, experiment group; C, control group; d, day; w, week; m, month; a, 6-month survival; b, 1-year survival; c, 2-year survival; d, KPS; e, ORR; f, ALT; g, AST; h, AFP; i, TCM symptom improvement; j, adverse events.

**TABLE 2:** Details of interventions.

First author and year	Formula	Ingredients of drastic medicinals	Control intervention
Zhang (2020)	Modified Sanjinchaihushusan decoction^△^	—	TACE (5-FU and OXA)
Shen L. N. (2020)	Modified Gexiazhuyu decoction^△^	—	TACE (5-FU, OXA, CF, ADM, and MMC)
Feng and Chen (2015)	Gexiazhuyu decoction^△^	—	TACE (5-FU, DDP, and ADM)
Cheng M. F. (2015)	Modified Yiganxiaogu decoction^△^	—	TACE (5-FU, DDP, and EPI)
Deng L. (2014)	Modified Jianpiyigan decoction^△^	—	TACE (5-FU, OXA, and MMC)
Liu X. (2013)	Modified WD-2 decoction^△^	—	TACE (5-FU, OXA, MMC, HCPT, DDP, and EPI)
Ji J. (2012)	Xiaoyaosan decoction^△^	—	TACE (5-FU, MMC, and DDP)
Li Y. H. (2011)	Chinese medicinals decoction^△^	—	TACE (5-FU, THP, and DDP)
Chi H. C. (2010)	Modified Shuganjianpi decoction^△^	—	TACE (5-FU, DDP, and EPI)
Wang A. M. (2020)	Modified Bazhen decoction^△^	—	TACE (EPI)
Song Y. N. (2017)	Modified Wenyangjiedu decoction^△^	—	TACE (CBP, THP, and MMC)
Ye W. D. (2015)	Modified Fupitiaogan decoction^△^	—	TACE
Huang J. Y. (2009)	Shenyi capsule and Cidan capsule^#^	*é zhú* (the dried rhizoma of *Curcuma kwangsiensis* S.G.Lee and C.F.Liang), *shān cí gū* (the dried pseudobulb of *Cremastra appendiculata* (D.Don) Makino), *mă qián zĭ* (the mature seed of *Strychnos nux-vomica* L.), *fēng fáng* (the nest of *Polistes olivaceous* (DeGeer)), *yā dăn zĭ* (the mature fruit of *Brucea javanica* (L.) Merr.), and *rén gōng niú huáng* (*Calculus Bovis Artifactus*)	TACE (5-FU, HCTP, DDP, and MMC)
Wang (2015)	Yangzhengxiaoji capsule^#^	*é zhú* (the dried rhizoma of *Curcuma kwangsiensis* S.G.Lee and C.F.Liang) and *tŭ biē chóng* (the dried insect body of *Eupolyphaga sinensis* Walker)	TACE (DDP, 5-FU, ADM, and MMC)
Rong Z. (2013)	Dujieqing oral liquid^#^	*wú gōng* (the dried insect body of *Scolopendra subspinipes mutilans* L. Koch), *bā jiăo lián* (the dried rhizoma and root of *Podophyllum versipelle* Hance), and *tŭ biē chóng* (the dried insect body of *Eupolyphaga sinensis* Walker)	TACE (DDP, GEM, and PYM)
Wang Q. M. (2016)	Ganxi tablet^#^	*zăo xiū* (the dried rhizome of *Paris polyphylla var. yunnanensis* (Franch.) Hand.-Mazz.)	TACE (arsenic trioxide)
Du H. P. (2018)	Biejiajian pill^#^	*tŭ biē chóng* (the dried insect body of *Eupolyphaga sinensis* Walker), *fēng fang* (the nest of *Polistes olivaceous* (DeGeer)), *qiāng lǎng* (the dried insect body of *Catharsius molossus* (Linnaeus)), *shǔ fù* (the dried insect body of *Armadillidium vurgare* (Latrelle)), and *xiāo shí* (Saltpetre)	TACE (5-FU, EPI, and HCPT)
Wang X. D. (2020)	Modified Fuzhengquxieyiai decoction^#^	*tŭ biē chóng* (the dried insect body of *Eupolyphaga sinensis* Walker) and *é zhú* (the dried rhizoma of *Curcuma kwangsiensis* S.G.Lee and C.F.Liang)	TACE (DDP and AZM)
Jiang R. R. (2020)	Fuhebiehua decoction^#^	*é zhú* (the dried rhizoma of *Curcuma kwangsiensis* S.G.Lee and C.F.Liang)	TACE (5-FU and OXA)
Tang Q. Y. (2015)	Modified Aitongxiao decoction^#^	*é zhú* (the dried rhizoma of *Curcuma kwangsiensis* S.G.Lee and C.F.Liang) and *sān léng* (the dried tuber of *Sparganium stoloniferum* (Buch.-Ham. ex Graebn.) Buch.-Ham. ex Juz.)	TACE (5-FU, THP, and MMC)
Ding R. F. (2012)	Modified Fuzheng decoction^#^	*é zhú* (the dried rhizoma of *Curcuma kwangsiensis* S.G.Lee and C.F.Liang)	TACE (OXA and GEM)
Zhang Q. (2007)	Gubenyiliu Ⅱ decoction^#^	*é zhú* (the dried rhizoma of *Curcuma kwangsiensis* S.G.Lee and C.F.Liang)	TACE (DDP, 5-FU, VDS, and EPI)
Zhang Z. Y. (2017)	Qingganhuayu decoction^#^	*é zhú* (the dried rhizoma of *Curcuma kwangsiensis* S.G.Lee and C.F.Liang) and *sān léng* (the dried tuber of *Sparganium stoloniferum* (Buch.-Ham. ex Graebn.) Buch.-Ham. ex Juz.)	TACE (5-FU, EPI, MMC, and DDP)
Li and Xu (2017)	Baoyuan decoction and Xiaoyao decoction^#^	*é zhú* (the dried rhizoma of *Curcuma kwangsiensis* S.G.Lee and C.F.Liang)	TACE (5-FU, THP, and DDP)

Abbreviations: 5-FU, 5-fluorouracil; DDP, cisplatin; OXA, oxaliplatin; CBP, carboplatin; CF, calcium folinate; ADM, doxorubicin; EPI, epirubicin; THP, pirarubicin; MMC, mitomycin; AZM, azithromycin; GZM, gemcitabine; PYM, bleomycin A5; HCPT, hydroxycamptothecin; VDS, vindesine sulfate. ^△^CMFs adopt the therapeutic principles of PSSQ. ^#^CMFs adopt the therapeutic principles of PCSQEP.

### Risk of Bias

Supplementary Figures S2, S3 showed the methodological quality of 24 trials. Eight trials applied a random number list. No studies described allocation concealment, blinding of participants and personnel, or blinding for outcomes, so their risks were unknown. All of the outcome data were complete, of which nine trials performed follow-up visits and one trial documented the reason for loss to follow-up and how these cases were handled. Two trials had poor adverse events reporting such that this outcome could not be incorporated into meta-analysis and were rated as high risk in the item of selective reporting. No clinical trial registration numbers were found for the remaining 22 studies, and the risk of selective reporting was unknown. Other risks of bias were also unclear.

### Primary Outcome

#### Survival Time

**FIGURE 2:**
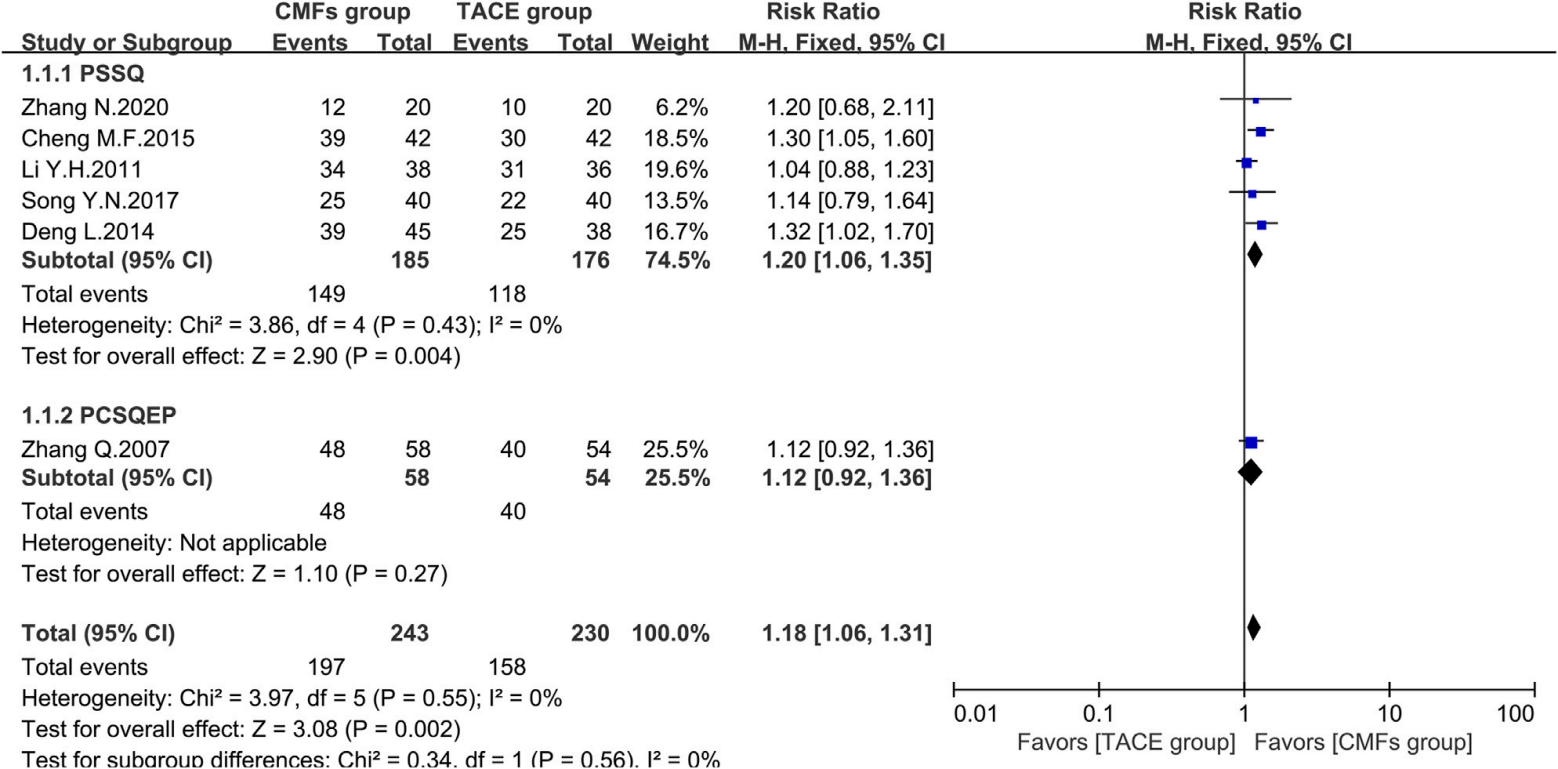
Effect of CMFs group versus TACE group on 6-month survival, including a comparison between PSSQ and PCSQEP in the subgroup.

**FIGURE 3:**
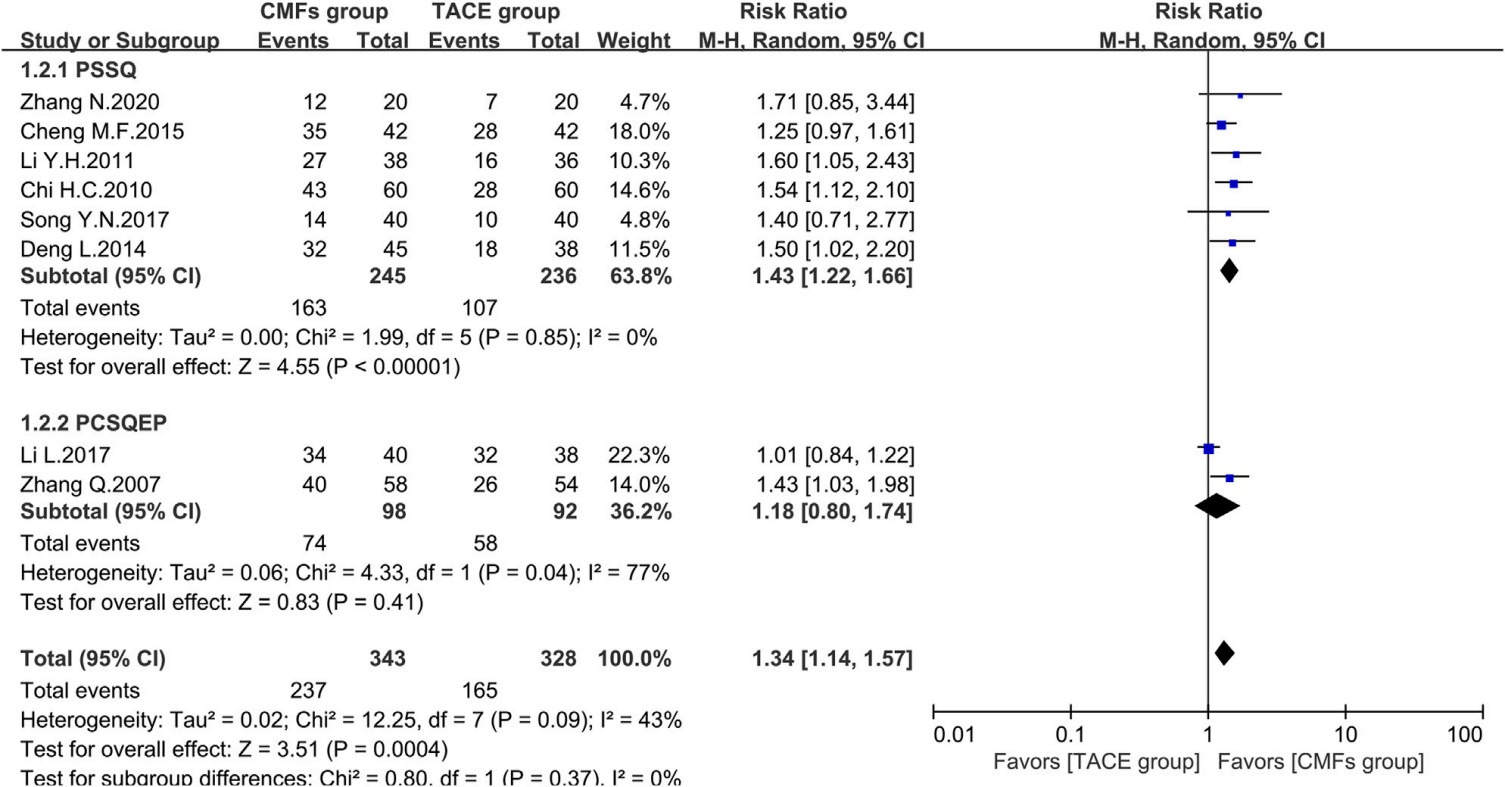
Effect of CMFs group versus TACE group on 1-year survival, including a comparison between PSSQ and PCSQEP in the subgroup.

**FIGURE 4:**
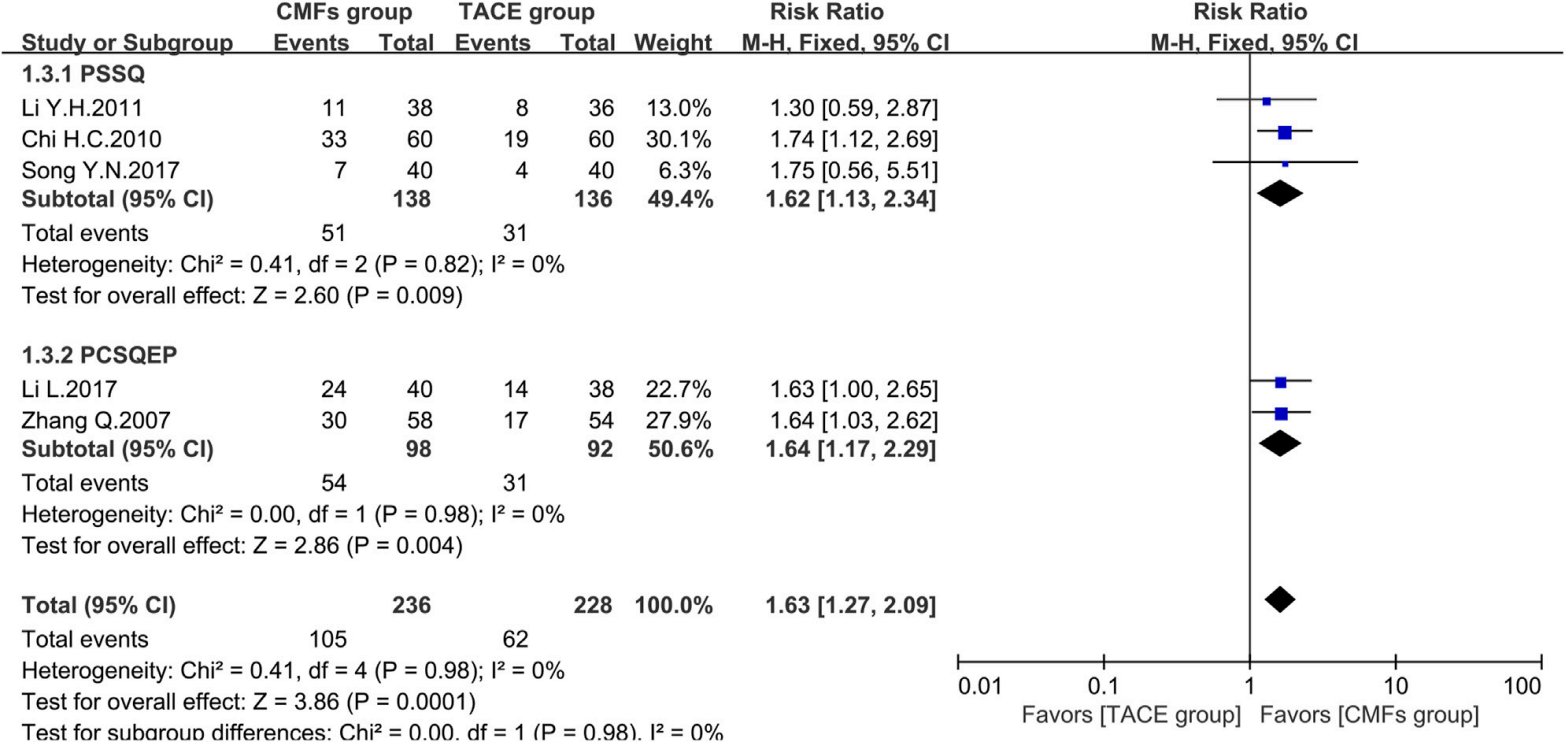
Effect of CMFs group versus TACE group on 2-year survival, including a comparison between PSSQ and PCSQEP in the subgroup.

A total of eight clinical trials reported the survival times of 6 months, 1 year, and 2 years of which the results were all shown by Figure 2, Figure 3, and Figure 4. Six trials reported 6-month survival rates [n = 473; RR = 1.18; 95%CI (1.06, 1.31); *p* = 0.002; I^2^ = 0%], with patients in the CMFs group having a higher survival rate than the TACE group. Subgroup analysis suggested that the trials complying with PSSQ improved the 6-month survival rate of patients in CMFs group [n = 361; RR = 1.20; 95%CI (1.06, 1.35); *p* = 0.004; I^2^ = 0%], while those trials complying with PCSQEP were not statistically significant [n = 112; RR = 1.12; 95%CI (0.92, 1.36); *p* = 0.27]. For the statistical analysis of 1-year survival, meta-analysis showed that the RR of 1-year survival was higher in the CMFs group than in the TACE group [n = 671; RR = 1.34; 95%CI (1.14, 1.57); *p* = 0.09; I^2^ = 43%]. In the subgroup analysis, the RR in the trials complying with PSSQ was higher in CMFs group [n = 481; RR = 1.43; 95%CI (1.22, 1.66); *p* = 0.85; I^2^ = 0%], while the trials complying with PCSQEP was not statistically significant [n = 190; RR = 1.18; 95%CI (0.80, 1.74); *p* = 0.04; I^2^ = 77%]. The sensitivity analysis showed that the study reported by Li L was the source of heterogeneity. CMFs also enhanced survival rate at 2 years [n = 464; RR = 1.63; 95%CI (1.27, 2.09); *p* = 0.0001; I^2^ = 0%]. In the subgroup analysis, trials complying with PSSQ [n = 274; RR = 1.62; 95%CI (1.13, 2.34); *p* = 0.009; I^2^ = 0%] and PCSQEP [n = 190; RR = 1.64; 95%CI (1.17, 2.29); *p* = 0.004; I^2^ = 0%] both reported that CMFs group performed better than TACE group.

### The Efficient Rate of Karnofsky Performance Status

**FIGURE 5:**
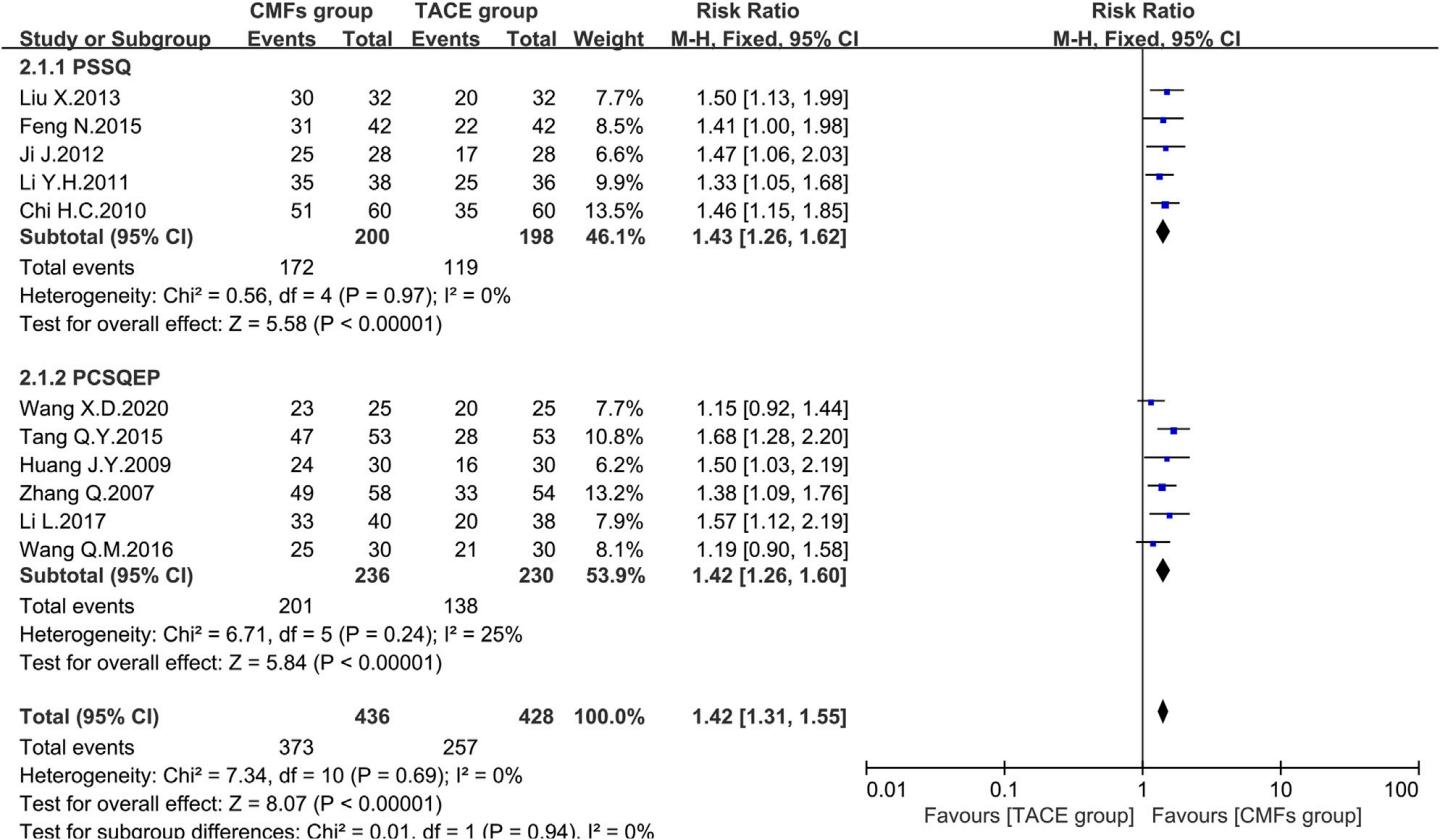
Effect of CMFs group versus TACE group on the efficient rate of KPS, including a comparison between PSSQ and PCSQEP in the subgroup.

According to the KPS defined by World Health Organization (WHO) (Palmer, 1982), the quality of life of patients whose postoperative KPS increased or decreased by no more than 10 points is considered stabilized or improved, which is called the efficient rate of KPS. The results of 11 trials showed that the CMFs group had a more significant improvement and stabilization effect on patients’ quality of life than the TACE group [n = 864; RR = 1.42; 95%CI (1.31, 1.55); *p* < 0.00001; I^2^ = 0%]. Concerning the subgroup analysis, five trials complying with PSSQ illustrated that the CMFs group enhanced the efficiency of KPS compared to the TACE group [n = 398; RR = 1.43; 95%CI (1.26, 1.62); *p* < 0.00001; I^2^ = 0%], which was similar to the result of remaining four trials in PCSQEP group [n = 466; RR = 1.42; 95%CI (1.26, 1.60); *p* < 0.00001; I^2^ = 25%] (Figure 5).

### Secondary Outcome

#### ORR

**FIGURE 6:**
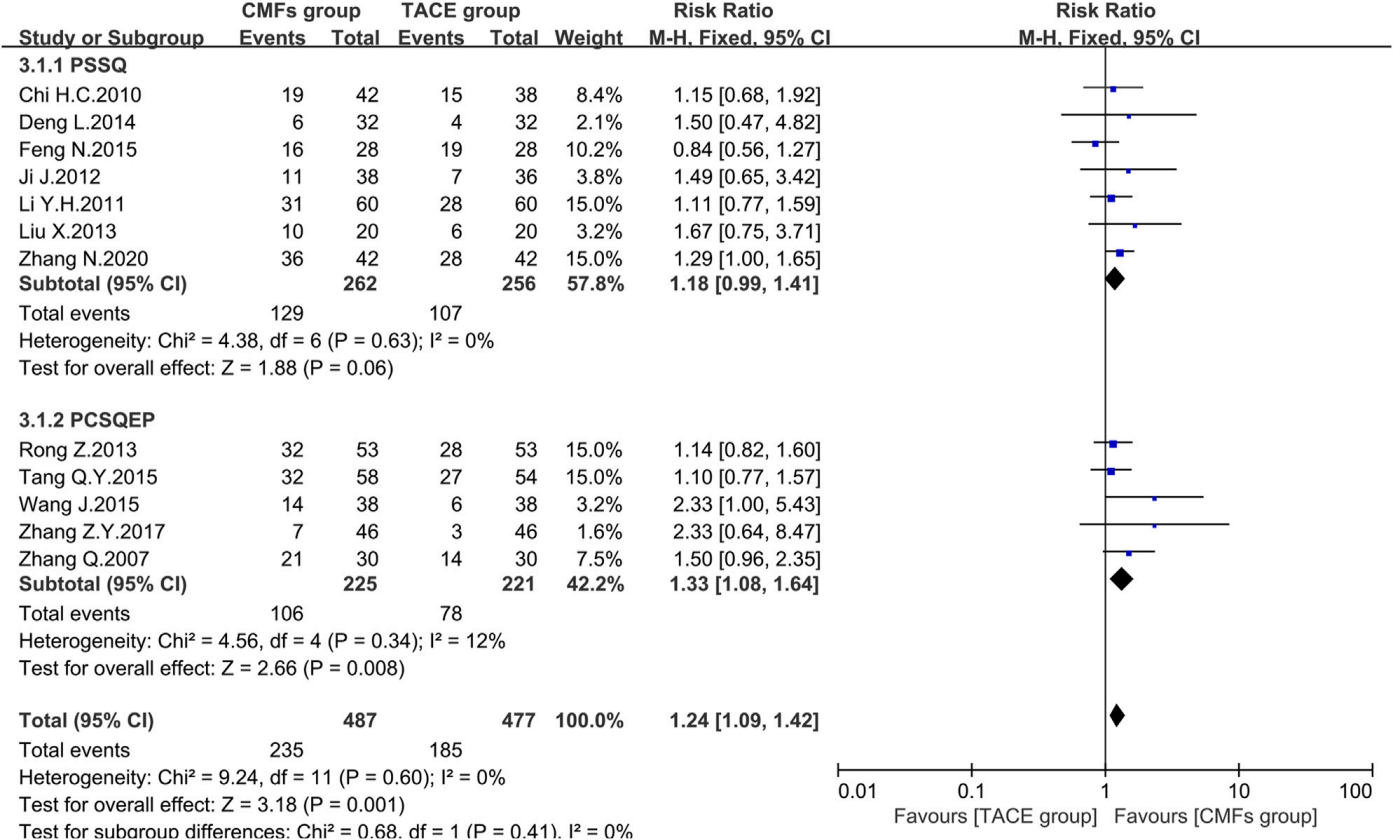
Effect of CMFs group versus TACE group on ORR applied the WHO criteria, including a comparison between PSSQ and PCSQEP in the subgroup.

**FIGURE 7:**
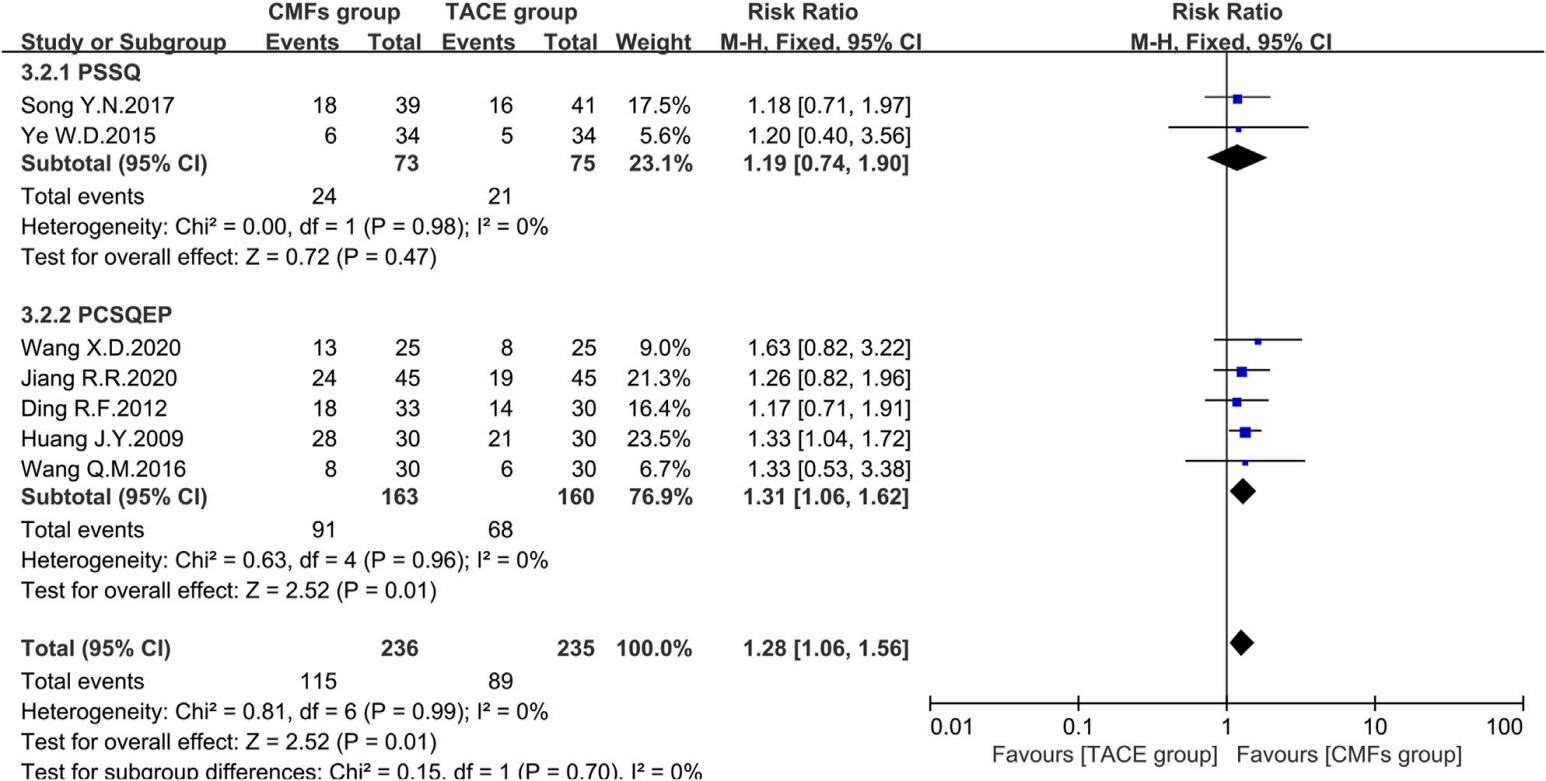
Effect of CMFs group versus TACE group on ORR applied the RECIST criteria, including a comparison between PSSQ and PCSQEP in the subgroup.

Nineteen clinical trials evaluated the efficacy of solid tumors with the benefit of two different evaluation criteria, the determination of overall response in solid tumors from WHO (Palmer, 1982), and response evaluation criteria in solid tumors (RECIST) (Therasse et al., 2000). ORR, the proportion of patients whose tumors shrank to a certain level and remained there for a certain period, was taken as an outcome indicator. A total of 12 clinical trials applied WHO criteria, and the results showed a more significant effect on reducing tumor size in the CMFs group than in the TACE group [n = 964; RR = 1.24; 95%CI (1.09, 1.42); *p* = 0.001; I^2^ = 0%]. RECIST criteria were applied to the remaining seven clinical studies and the results were consistent with the former [n = 471; RR = 1.28; 95%CI (1.06, 1.56); *p* = 0.01; I^2^ = 0%]. In the subgroup analysis, none of the trials complying with PSSQ were statistically significant using either the WHO criteria [n = 518; RR = 1.18; 95%CI (0.99, 1.41); *p* = 0.06; I^2^ = 0%] or the RECIST criteria [n = 148; RR = 1.19; 95%CI (0.74, 1.90); *p* = 0.47; I^2^ = 0%]. However, the trials complying with PCSQEP showed that CMFs were able to remarkably improve the ORR in WHO criteria [n = 446; RR = 1.33; 95%CI (1.08, 1.64); *p* = 0.008; I^2^ = 12%] and RECIST criteria [n = 323; RR = 1.31; 95%CI (1.06, 1.62); *p* = 0.01; I^2^ = 0%] (Figure 6 and Figure 7).

#### ALT

**FIGURE 8:**
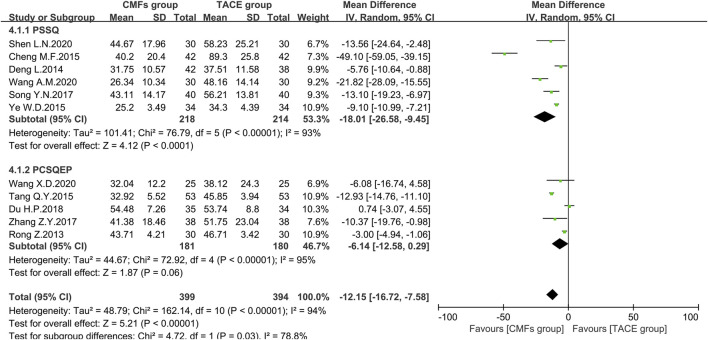
Effect of CMFs group versus TACE group on ALT, including a comparison between PSSQ and PCSQEP in the subgroup.

**FIGURE 9:**
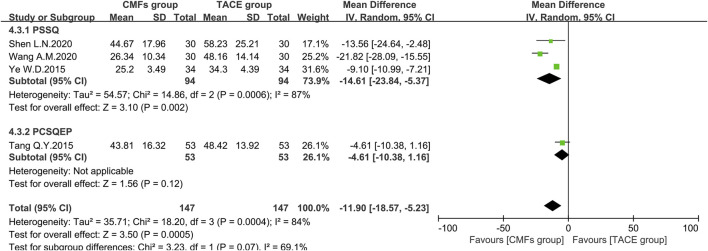
Effect of CMFs group versus TACE group on ALT after treated with TACE for 4 weeks, including a comparison between PSSQ and PCSQEP in the subgroup.

A sum of 11 articles reported the effect of the combination of CMFs and TACE on ALT levels; see Figure 8. Overall, ALT levels were significantly reduced after treatment with CMFs [n = 793; MD = −12.15; 95%CI (−16.72, −7.58); *p* < 0.00001; I^2^ = 94%]. The result of six trials complying with PSSQ reported beneficial results for the CMFs group [n = 432; MD = −18.01; 95%CI (−26.58, −9.45); *p* < 0.00001; I^2^ = 93%], while the five trials complying with PCSQEP showed no statistical significance [n = 361; MD = −6.14; 95%CI (−12.58, 0.29); *p* < 0.00001; I^2^ = 95%]. In addition, the effects of the PCSQEP group changed with the use of different effect models, which proved that the efficacy of trials complying with PCSQEP was unclear. As both subgroup and overall analyses exhibited a large heterogeneity, a random-effects model was used for the analyses. We speculated that the inconsistent timing of postoperative evaluation of ALT may be contributing to the heterogeneity. Therefore, four trials meeting the requirements that measured ALT after 4 weeks of TACE were analyzed, as shown in Figure 9. Similar to the results of those studies that did not limit the evaluation time, the CMFs [n = 294; MD = −11.90; 95%CI (−18.57, −5.23); *p* = 0.0005; I^2^ = 84%] were able to reduce ALT levels. Trials complying with PSSQ [n = 188; MD = −14.61; 95%CI (−23.84, −5.37); *p* = 0.002; I^2^ = 87%] achieved the same results. Interestingly, trials complying with PCSQEP group showed no statistical significance between CMFs group and TACE group [n = 106; MD = −4.61; 95%CI (−10.38, 1.16); *p* = 0.12]. Since studies are heterogeneous, a random-effects model was selected. Notably, the study of Wang A. M was a source of heterogeneity because excluding her study resulted in an I^2^ = 0% for the PSSQ group and an I^2^ = 29% overall.

#### AST

**FIGURE 10:**
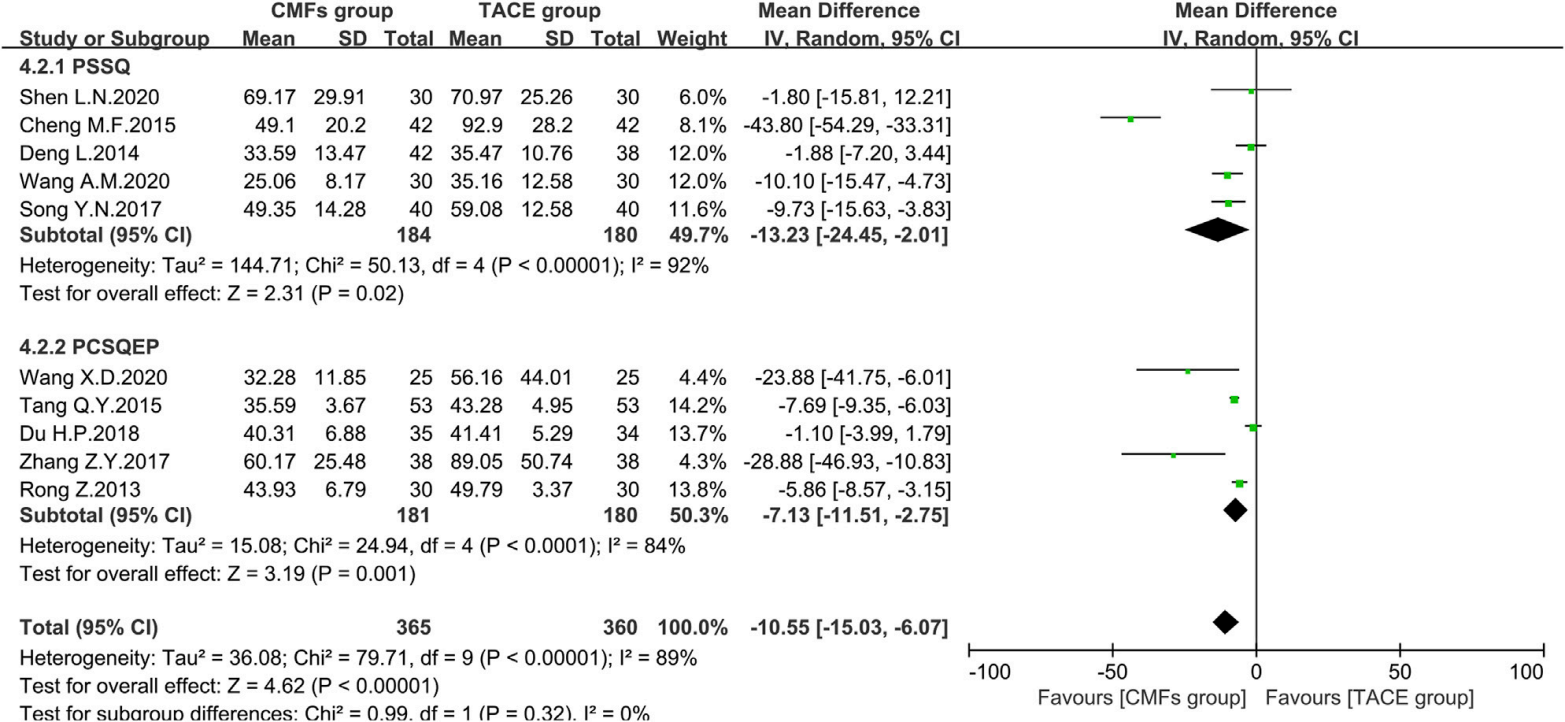
Effect of CMFs group versus TACE group on AST, including a comparison between PSSQ and PCSQEP in the subgroup.

**FIGURE 11:**
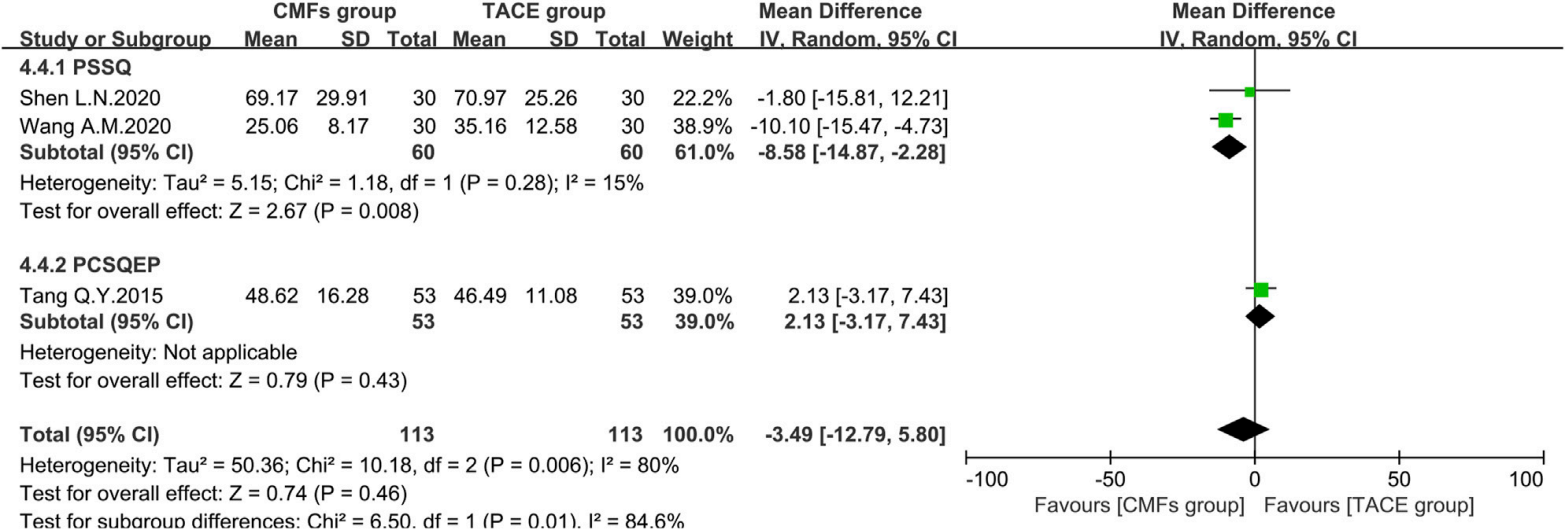
Effect of CMFs group versus TACE group on AST after treated with TACE for 4 weeks, including a comparison between PSSQ and PCSQEP in the subgroup.

Ten articles reported the effects of the combination of CMFs and TACE on AST levels, and the results are shown in Figure 10. Owing to considerable heterogeneity, a random-effects model was conducted for meta-analysis. The CMFs group was able to reduce AST levels compared to TACE group [n = 725; MD = −10.55; 95%CI (−15.03, −6.07); *p* < 0.00001; I^2^ = 89%]. Similarly, trials complying with PSSQ [n = 364; MD = −13.23; 95%CI (−24.45, −2.01); *p* < 0.00001; I^2^ = 92%] and PCSQEP [n = 361; MD = −7.13; 95%CI (−11.51, −2.75); *p* < 0.0001; I^2^ = 84%] both suggested that CMFs group achieved better reduction of AST levels. The reasons for the high heterogeneity of AST results are the same as ALT. There were three clinical studies in which AST was measured 4 weeks after TACE, and the results were shown in Figure 11. Since I^2^ = 80% for the three articles, a random-effects model was still used. The overall efficacy was not statistically significant between CMFs group and TACE group [n = 226; MD = −3.49; 95%CI (−12.79, 5.80); *p* = 0.46; I^2^ = 80%], and trials complying with PSSQ demonstrated a diminution in AST in the CMFs group [n = 120; MD = −8.58; 95%CI (−14.87, −2.28); *p* = 0.008; I^2^ = 15%], while PCSQEP was also nonstatistically significant [n = 106; MD = 2.13; 95%CI (−3.17, 7.43); *p* = 0.43]. Nevertheless, after applying a fixed-effects model, the overall AST outcome was statistically significant.

#### AFP

**FIGURE 12:**
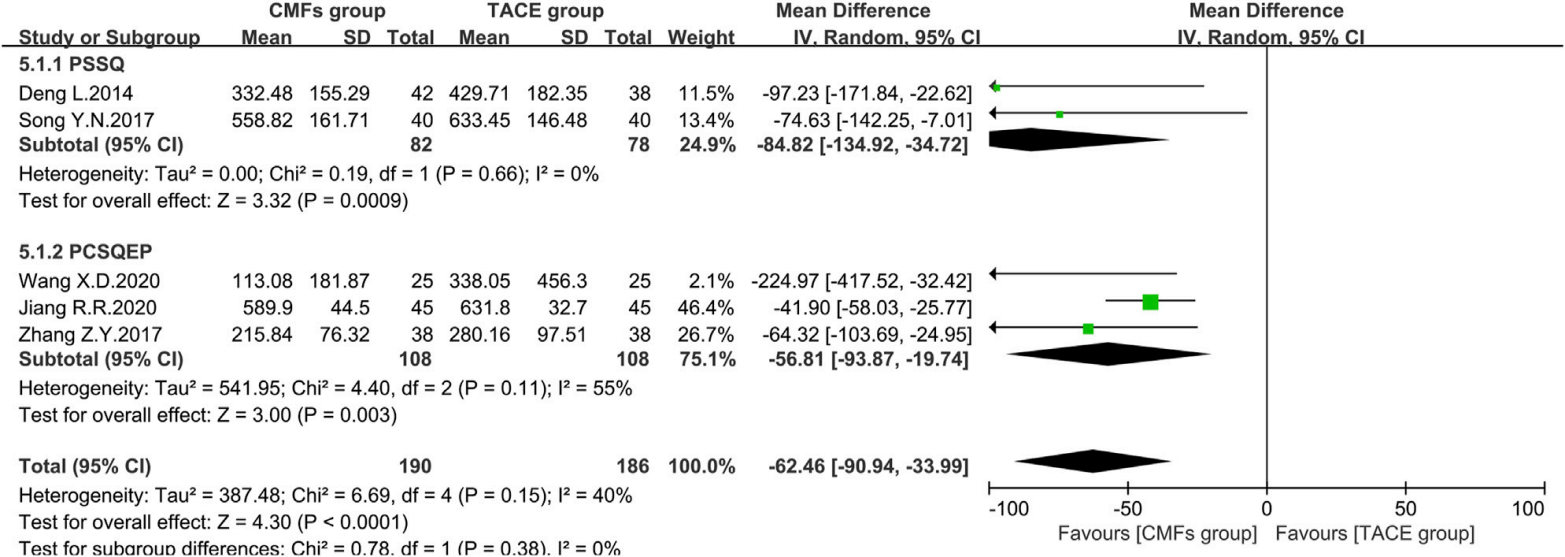
Effect of CMFs group versus TACE group on AFP, including a comparison between PSSQ and PCSQEP in the subgroup.

Eight clinical trials with AFP outcomes were reported, five of which were included in the meta-analysis. The remaining three could not be pooled because one clinical trial reported by Wang J (Wang, 2015) used units of iu/ml, one was dominated by Rong Z (Rong et al., 2013), which evaluated patients with AFP <400 ng/ml and >400 ng/ml separately, and one used categorical data (Li et al., 2011). The results of the remaining studies included in the meta-analysis were presented in Figure 12. The CMFs group was able to reduce AFP levels in comparison to the TACE group [n = 376; MD = -62.46; 95%CI (−90.94, −33.99); *p* < 0.0001; I^2^ = 40%], and similar results were obtained in the trials complying with PSSQ [n = 160; MD = −84.82; 95%CI (−134.92, −34.72); *p* = 0.0009; I^2^ = 0%] and PCSQEP [n = 216; MD = −56.81; 95%CI (−93.87, −19.74); *p* = 0.003; I^2^ = 55%]. A random-effects model was applied regarding the I^2^ > 50% in the PCSQEP group. Wang X. D’s study was a source of heterogeneity, and the outcome of trials complying with PCSQEP was invariant after this trial exclusion (Wang et al., 2020). In Wang J’s study, the decrease in AFP was more pronounced in the CMFs group with the addition of drastic medicinals [n = 92; MD = −10.93; 95%CI (−16.10, −5.76); *p* < 0.0001] (Wang, 2015). In Rong Z’s study, the decrease in AFP after the combination of CMFs and TACE was not statistically significant in patients with AFP < 400 ng/ml before treatment, while it was significant in patients with AFP > 400 ng/ml [n = 22; MD = 14.12; 95%CI (−67.96, 96.20); *p* = 0.74]. In Li Y. H’s study (Li et al., 2011), the improvement rate of AFP was 26.3% in the CMFs group and 22.2% in the TACE group (Rong et al., 2013).

#### The Improvement Rate of Symptoms

**FIGURE 13:**
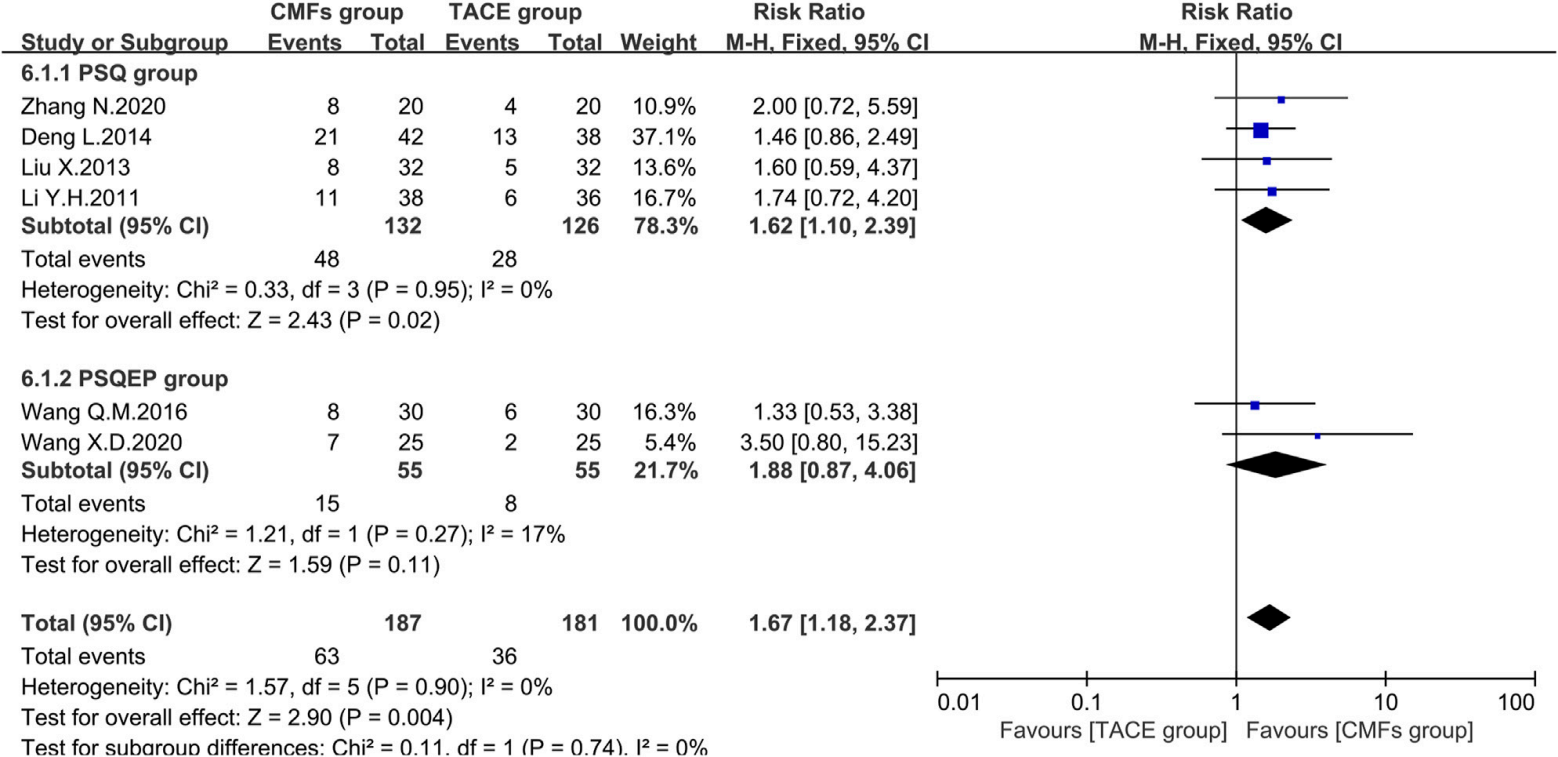
Effect of CMFs group versus TACE group on the improvement of symptoms, including a comparison between PSSQ and PCSQEP in the subgroup.

Referring to Guidelines of Clinical Research of New Drugs of Traditional Chinese Medicine, patients with hepatic cancer would have typical symptoms such as liver pain, abdominal distension, belching, and bitterness in the mouth. The authors of six trials scored according to the severity of the symptoms before treatment and then rated a significant improvement if the score after treatment dropped > 3/2. The meta-analysis results of symptom improvement rate were shown in Figure 13. In general, the improvement rate of symptoms in the CMFs group was higher than that in the TACE group [n = 368; RR = 1.67; 95%CI (1.18, 2.37); *p* = 0.004; I^2^ = 0%]. In the subgroup analysis, the trials complying with PSSQ revealed that CMFs could increase the number of people whose symptom score decreased by more than 3/2 compared with the TACE group [n = 258; RR = 1.62; 95%CI (1.10, 2.39); *p* = 0.02; I^2^ = 0%]. However, the result of trials complying with PCSQEP had no statistically significant [n = 110; RR = 1.88; 95%CI (0.87, 4.06); *p* = 0.11; I^2^ = 17%].

### Adverse Events

A total of 13 studies reported adverse events after the combination of CMFs and TACE treatment. The trial of Wang X.D.2020 (Wang et al., 2020) reported that no adverse events occurred in the included patients, and the study of Liu X.2013 (Liu et al., 2013) and Ji J.2012 (Ji et al., 2012) did not provide enough adverse events data for meta-analysis. The remaining 10 studies adopted different evaluation standards; for example, some studies (Zhang et al., 2007; Chi et al., 2010; Ye et al., 2015; Wang et al., 2016) adopted the assessment of acute and subacute toxic effects from WHO (Palmer, 1982), and Ding R. F’s research (Ding R.F., 2012) adopted the Common Terminology Criteria for Adverse Events version 3.0 (CTCAE) (Trotti et al., 2003). Therefore, we merged the data to be comparative and representative and presented it in Supplementary Table S4. As long as there was I^2^ > 50% in the subgroup or the overall, the random-effects model was employed for analysis. Three trials reported the overall incidence of adverse events with nonstatistically significant results. In terms of fever, total hepatotoxicity incidence, neurotoxicity, total leukocyte decline rate, all grades of platelet decline, and hemoglobin decline, there were no statistically significant in both overall and subgroups. However, CMFs played a role in alleviating nausea and vomiting and bone marrow suppression whether the result of subgroup or overall supported this finding. In addition, patients with inappetence and gastrointestinal symptoms exhibited less incidence in the CMFs group. Although the CMFs group was not statistically significant in improving the toxicity of chemotherapy to platelets and hemoglobin, trials complying with the PCSQEP group or PSSQ showed the improvement in CMFs was superior to TACE. It was worth noting that some studies have graded the toxicity of adverse effects by severity, such as gastrointestinal symptoms, fever, hepatotoxicity, and leukocyte decline. CMFs group was not as good as the TACE group or was not statistically significant in relieving the above symptoms of mild degrees, but it could reduce serious adverse events. The results for the WBC decline and digestive symptoms Ⅰ were unstable. After switching the effect model, the CMFs group would increase the incidence of digestive symptoms Ⅰ and WBC levels compared with the TACE group.

### Publication Bias

A funnel plot analysis of the KPS efficiency rate in the primary outcome was conducted because the number of trials for the survival outcome was too small and the power of the test was insufficient to assess symmetry (Higgins et al., 2019). For this reason, the method proposed by Harbord was used to explore the publication bias in survival rates, which was performed in Stata 16. The result shown in Supplementary Figures S4, S5, and S7 indicated that there was no publication bias for the KPS efficiency rate and 6-month and 2-year survival. Publication bias was found in the 1-year survival rate (see Supplementary Figure S6).

## Discussion

TACE can only be used as a palliative treatment for patients. Although the survival time could be prolonged after accepting TACE treatment, the median survival time reported in the previous study was only 21 months (Yang et al., 2019). Moreover, the side effects always appear when receiving TACE treatment, such as vomiting, hair loss, and sensory disturbances. The endpoint of antitumor treatment is emphasized with providing the longest survival and less impaired quality of life (Bruix et al., 2015). Actually, CMFs could prolong survival time corresponding with improving the quality of life of cancer patients by the combination of multiple compounds. At present, the research on the antitumor mechanism of TCM mainly focuses on several ways: 1) Inhibit tumor proliferation and migration. An effective anticancer component β-elemene extracted from *Curcumae Rhizoma* could inhibit HepG2 cell proliferation, induce cell cycle arrest, and mediate apoptosis by regulating topoisomerase, microtubular polymerization, and FasL (Zhai et al., 2019). 2) Improve immunity and general condition. Various herbal compounds have been shown to have positive effects on the immune system. For example, ginsenoside Rg3 could promote the differentiation of naive T cells to Th1 cells in LC mice by activating DCs (Wang et al., 2020). Cachexia is a common syndrome in patients with intermediate-advanced malignant tumors. Imperatorin inhibits STAT3 phosphorylation by directly binding to the SH2 domain of STAT3, which can reduce muscle wasting (Chen et al., 2020). 3) Reduce the side effects of chemotherapy drugs in the body, especially to protect normal cells and tissues in the body from damage caused by chemotherapy or radiotherapy. Cell death caused by damaging DNA is a therapeutic target of anticancer treatment and also a trigger for the occurrence of side effects in patients. It has been reported that rocaglamide could reduce DNA damage-induced toxicity in nonmalignant cells by inhibiting the p53 pathway (Becker et al., 2014). 4) Reduce resistance to chemotherapeutic agents in order to proceed to the next chemotherapeutic intervention as soon as possible. Astragaloside II was found to sensitize human hepatocellular carcinoma cells to 5-FU by inhibiting cellular autophagy involved in the MAPK-mTOR signaling pathway (Wang et al., 2017). Hence, as an adjuvant therapy, the antitumor treatment of CMFs is very promising.

In this study, the survival rate of PLC patients within 6 months, 1 year, and 2 years was enhanced, and so does the KPS. In addition, the scores of typical LC symptoms, including flank pain, belching, depression, and bitter mouth, were reduced through CMFs treatment. This evidence demonstrated that the combination of CMFs and TACE could improve the quality of life of patients. Assessment of solid tumor responses has proved to be a surrogate index for survival (Sieghart et al., 2015). Based on the relevant assessing standard followed by the RECIST or/and WHO, the tumor lesions were markedly reduced or even disappeared after using CMFs. AFP is also another classic tumor marker, which is mainly used for predicting advanced disease and poor prognosis (Bruix et al., 2015). A meta-analysis including five RCTs and three clinical trials of descriptive statistics indicated an apparent decrease of AFP after the treatment of CMFs. This result suggests that CMFs had a positive effect on tumor marker, which was consistent with the finding of Cheng Y. (Cheng et al., 2020). Generally speaking, CMFs could prolong the survival rate, ameliorate the quality of life, diminish solid tumors, and lower tumor markers in PLC patients at the intermediate-advanced stage. It is interesting that the utilization of traditional, complementary, and alternative medicine is as high as 80% in less developed countries according to WHO report (Hill et al., 2019). The finding in this meta-analysis could support that CMFs as an anticancer treatment had a good application prospect, which contributed to reducing the cost of treatment for cancer patients in low-income countries with obtaining the same medical benefits.

The drug caused ischemic necrosis of the tumor tissue along with transient or chronic liver injury after treating TACE (Miksad et al., 2019). Moreover, the level of AST/ALT ratio is also closely related to liver necrosis, and it may aggravate the hepatic neoplasm invasion (Zhang et al., 2019). Thus, liver function is necessary to evaluate. However, there existed a great heterogeneity in ALT and AST outcomes. Based on the knowledge for this disease, the heterogeneity resulted from five aspects, such as the liver function of patients before treatment, the type and dosage of drugs used in TACE, the course of TACE, the evaluation time after TACE treatment, and the liver toxicity of TCM.

Firstly, the total heterogeneity of ALT becomes I^2^ = 92% (n = 7) when removing trials that involved patients with Child-Pugh C or did not record the Child-Pugh level of patients. The total heterogeneity of AST becomes I^2^ = 77% (n = 8), with no heterogeneity being changed after liver function unified. Secondly, the types of chemotherapeutic drugs used in clinical trials were different. Simultaneously, some studies emphasized that the dosage of chemotherapeutic drugs would be adjusted according to the patient’s tumor size, hepatorenal function, and general conditions (Deng et al., 2014; Zhang et al., 2017; Shen et al., 2020), which rendered the inconsistent use of chemotherapeutic drugs. Lack of standardized TACE treatment protocols is also a current problem (Sieghart et al., 2015). Besides, cTACE and DEB-TACE have different toxicity on the liver. Some studies have shown that the drug-related systemic and liver toxicity of the DEB-TACE group was pronouncedly turned down, comparing with the cTACE group (Sieghart et al., 2015). While other studies have illustrated, DEB-TACE increased the risk of liver-related damage (Raoul et al., 2019). More rigorous RCTs are required to prove the severity of the liver injury of DEB-TACE and cTACE. Thirdly, the duration and interval of TACE are also essential elements affecting liver function. And the successful efficiency of TACE can be reflected by the evaluation of tumor response (Sieghart et al., 2015). However, it is sporadic to achieve the goal of treatment in a single course of TACE treatment. Therefore, the Assessment for Retreatment with TACE (ART) score, tumor size, tumor number, AFP level, Child-Pugh class, Objective Response after TACE (SNACOR) model, AFP level, BCLC stage, Child-Pugh class, Response after TACE (ABCR score), and Hepatoma Arterial-embolization Prognostic (HAP) score are the reference tools used by doctors to assess whether a patient can undergo TACE again (Chon et al., 2019). So far, there is an absence of high-quality evidence to prove the optimal interval between two TACE treatments. Shortening the interval between two TACE treatments may result in hepatic dysfunction or liver failure (Intaraprasong et al., 2016). Meanwhile, there is a greater chance of liver failure after repeating TACE treatments (Raoul et al., 2011). Fourthly, transient liver injury generally takes 1 month to recover after TACE (Barzakova et al., 2019; Miksad et al., 2019). Accordingly, those clinical trials that evaluated liver function after being treated with TACE for 4 weeks were selected to assess its effect on ALT and AST. When conducting sensitive analysis on selected trials, the RCT performed by Wang A.M (Wang et al., 2020) brought a significant heterogeneity on ALT and AST. Before removing this trial, the overall heterogeneity remained significant, and the impact of CMFs on AST was not statistically significant. Finally, drug-induced liver injury (DILI) caused by herbal medicine is the potential aspect leading to heterogeneity (Andrade et al., 2019). Herbs contain thousands of chemical compounds. Some compounds have been proved to be hepatotoxins including volatile compounds, phytotoxic proteins, glycosides, terpenoid lactones, terpenoids, alkaloids, anthraquinones, and phenolic acids (Quan et al., 2020). However, it is unclear that if the proper application of formulas containing these medicinals contributes to liver injury or not. Among the included trials, the longest treating period with CMFs is 3 months, during which the toxic chemical components may continually accumulate in the liver and eventually damage the liver cell.

Holism is a major feature of the TCM theoretical system. TCM holds the point that disease is by the imbalance of the body (So et al., 2019). TACE is considered a local treatment, which can cause ischemic necrosis of neoplasm and eliminate the pathogenic factors. The CMFs emphasize synergism and detoxification through the combination of chief medicinal, deputy medicinal, assistant medicinal, and envoy medicinal (Wei et al., 2018). CMFs are characterized by multicomponent and multitarget effects (Luan et al., 2020). In the treating practice, in order to treat simultaneously both the whole body and the local lesions, TCM physicians often adopt PCSQEP with drastic medicinals and Qi replenishing medicinals in their prescriptions.

LC was stubbornly intertwined with phlegm, blood stasis, and toxin, so drastic medicinals accurate in intervening cancer cells were frequently applied to eliminate pathogens. Pharmacological experiments demonstrated that drastic medicinals can fight against tumors (Yoon et al., 2018; Chang et al., 2020; Jia et al., 2021), promote blood circulation, and curb pain caused by oxaliplatin. However, the evidence of clinical studies was inadequate to explain that drastic medicinals had a strong tumor-suppressive effect and its rationality in clinic usage. It is worth noting that excessive usage of drastic medicinals would be emerging resulting by the physician differentiate the syndrome of the disease improperly, which would further damage the healthy Qi, disrupt the balance of homeostasis, and ultimately promote the development of the disease. In terms of the therapeutic effect of TACE, the necessity for applying the combination of drastic medicinals and TACE on PLC needs to be urgently evaluated.

Depending on the properties and dosage of the medicinals, the drastic medicinals are generally divided into three categories. The first is the medicinals with an obvious drug bias, such as the bitter-cold medicinal *bái huā shé shé căo* (the whole plant of *Scleromitrion diffusum* (Willd.) R.J.Wang); the second is high-dose medication (generally more than 50 g); the third is toxic medicinals and overactive medicinals, which are mostly used in malignant diseases. It is worth clarifying that only the third category meets the entry requirements in this work. The clinical trials that applied drastic medicinals are believed to follow the PCSQEP.

The results displayed that trials complying with PSSQ or PCSQEP could improve the 2-year survival rate, stabilize or increase the KPS score, and decrease the AFP level. Interestingly, the CMFs group following PSSQ could improve the survival rate of 6 months and1 year and the curative effect of TCM symptoms, but the PCSQEP group had no statistical significance for these outcomes. There was no statistically significant ORR for the RECIST and WHO criteria in trials complying with PSSQ, while the trials complying with PCSQEP displayed a remarkable improvement. In particular, the survival rate reflected the long-term impact of treatment, and the solid tumor response reflected the direct effect of the drug on the tumor, which is a manifestation of the short-term impact. PSSQ and PCSQEP show different efficacy in long-term and short-term impact, which may be related to PLC patients’ weak physical status. The reason why CMFs following PSSQ achieved better efficacy in the long term may stem from Qi replenishing medicinals with the effects of immunity-boosting and energy-supplementing (Wang et al., 2017). However, following PCSQEP, the cytotoxic effects of TACE and drastic medicinals synergistically fighting cancer were too intense to keep the normal life activities of the body for a long time. Perhaps by adjusting the compatibility of CMFs or using the two therapeutic regimens at intervals, it is possible to achieve the goal of improving both the short-term and the long-term curative effect of middle to advanced PLC. Notably, few studies adopted PCSQEP treatment regimens and followed up for 6 months or more, so the results in the subgroup analysis should be treated with caution.

Postembolism syndrome is reported as a typical adverse reaction of TACE treatment. Meanwhile, some Chinese medicinals applied in CMFs may cause adverse events. It is unclear whether the therapeutic effect of CMFs is greater than its damage due to the key role of liver metabolism. This study indicated that the entire adverse events of the combination of CMFs and TACE treatment were uncertain, but the reductions of nausea and vomiting, inappetence, digestive symptoms, and bone marrow suppression were clear. Interestingly, the combination of CMFs and TACE increased the risk of certain mild adverse events but reduced the incidence of serious adverse events. This suggests that physicians needed to be cautious when using Chinese medicinals in clinical practice, especially in East Asia, where herbal medicine is prevalent and chronic hepatitis B virus infection is the main reason for the high incidence of HCC patients (Ashtari et al., 2015). Studies have shown that, in a large number of DILI populations, 10% have preexisting liver disease. Besides this, slow acetylator genotype in East Asian ancestry would cause accumulation of toxic components in the body and aggravate the adverse events (Andrade et al., 2019). From another perspective, mild adverse events would gradually disappear with routine expectant treatment. On the contrary, the drop of serious adverse events reflected the therapeutic effect of CMFs that enhanced the patients’ quality of life to a certain extent and prepared them for the next TACE operation. Drastic medicinals were known to be toxic, and in this meta-analysis, they did not have a statistically significant influence on the overall occurrence of adverse events. Although we were particularly concerned about the incidence of various adverse events of drastic medicinals, due to the few included studies, the current results only showed that drastic medicinals had to alleviate effects on nausea and vomiting, digestive symptoms, and bone marrow suppression. More evidence is needed to support whether drastic medicinals will aggravate the burden of liver function in PLC patients and cause more serious adverse events.

The methodological quality of RCTs of TCM treatment is generally low and has been criticized for a long time (Li et al., 2014). In the full-text screening, several trials did not detail the randomization generation and no studies were blinded to the researchers and patients. Most of the interventions in the included studies were decoctions, but it was impractical to use a placebo of decoctions because it was challenging to design placebos to smell, look, and taste like a medicinal liquid in fact (Zhong et al., 2010). Therefore, the restrictive conditions of the experimental design were established to promote the credibility and authenticity of this study consulting the approach of RCTs (Supplementary Table S1).

Trials with diagnostic criteria, inclusion criteria, and exclusion criteria were regarded as stringency and reliability and the comparable baseline information was suitable for accurately evaluating the efficacy of the interventions. At the same time, the washout period of included studies could exclude the interference of antecedent treatments on TACE and TCM treatment. The remaining studies were from journals and were more authentic after review, which could also ensure the reliability of the conclusions in this study and the quality of the whole article.

## Conclusion

This meta-analysis confirmed that the combination of CMFs and TACE treatment ameliorated the primary outcome, survival time, KPS, and secondary outcomes, ORR, AFP, and TCM symptoms in PLC patients at the intermediate-advanced stage. CMFs reduced some adverse events after TACE but may increase the occurrence of mild adverse events. Moreover, adopting PCSQEP was shown to be beneficial to patients in the short-term effect, while PSSQ made the patient benefit more in the long-term impact. Appropriate reduction drastic medicinals use or reasonable choice PCSQEP and PSSQ treating methods may prolong the lifespan of patients. As is well known, the meta-analysis aims to evaluate the efficacy and safety of PSSQ and PCSQEP in PLC. The research reveals that the anticancer effect with drastic medicinals not only comes from TCM theory and experimental research but also was supported by evidence-based medicine. Due to the limited quality and a small number of included studies, the results of this meta-analysis still needed to be kept with caution and required validation by more high-quality studies in the future.

## Data Availability

The original contributions presented in the study are included in the article/Supplementary Material; further inquiries can be directed to the corresponding author.
