# Corrigendum: The Therapeutic Principle of Combined Strengthening Qi and Eliminating Pathogens in Treating Middle-Advanced Primary Liver Cancer: A Systematic Review and Meta-Analysis

**DOI:** 10.3389/fphar.2022.846668

**Published:** 2022-02-04

**Authors:** Yingqi She, Qinfeng Huang, Zhen Ye, Yu Hu, Mingquan Wu, Kaihua Qin, Ailing Wei, Xin Yang, Yuyao Liu, Cuihan Zhang, Qiaobo Ye

**Affiliations:** ^1^ School of Basic Medical Sciences, Chengdu University of Traditional Chinese Medicine, Chengdu, China; ^2^ Department of Oncology, The First Affiliated Hospital, Guangxi University of Chinese Medicine, Nanning, China; ^3^ Department of Pharmacy, Sichuan Provincial Orthopedic Hospital, Chengdu, China; ^4^ Health Preservation and Rehabilitation College, Chengdu University of Traditional Chinese Medicine, Chengdu, China; ^5^ Department of Liver Disease, The First Affiliated Hospital, Guangxi University of Chinese Medicine, Nanning, China; ^6^ Pharmacy College, Chengdu University of Traditional Chinese Medicine, Chengdu, China

**Keywords:** Chinese medicinal formulas, transcatheter arterial chemoembolization, drastic medicinals, therapeutic principle, primary liver cancer, meta-analysis

In the published article, there was an error in **affiliation 2.** Instead of “Department of Oncology, The First Affiliated Hospital, Guangxi University of Chinese Medicine, Chengdu, China”, it should be “Department of Oncology, The First Affiliated Hospital, Guangxi University of Chinese Medicine, Nanning, China”.

The authors apologize for this error and state that this does not change the scientific conclusions of the article in any way. The original article has been updated.

