# Supplementary material for: The Therapeutic Principle of Combined Strengthening Qi and Eliminating Pathogens in Treating Middle-Advanced Primary Liver Cancer: A Systematic Review and Meta-Analysis
Source: Front Pharmacol. 2021 Oct 27;12:714287. doi: 10.3389/fphar.2021.714287 (PMC8578139; doi:10.3389/fphar.2021.714287)
Supplement: Supplementary file 1 [file DataSheet2.PDF]

Supplementary Figure legends

Figure S1: The flowchart of meta analysis.

Figure S2: Risk of bias graph.

Figure S3: Risk of bias summary.

Figure S4: The funnel plot of the efficient rate of KPS.

Figure S5: The publication bias of 6 months survival time.

Figure S6: The publication bias of one year survival time.

Figure S7: The publication bias of two years survival time.
