# Supplementary figures and images for: The Therapeutic Principle of Combined Strengthening Qi and Eliminating Pathogens in Treating Middle-Advanced Primary Liver Cancer: A Systematic Review and Meta-Analysis
Source: Front Pharmacol. 2021 Oct 27;12:714287. doi: 10.3389/fphar.2021.714287 (PMC8578139; doi:10.3389/fphar.2021.714287)

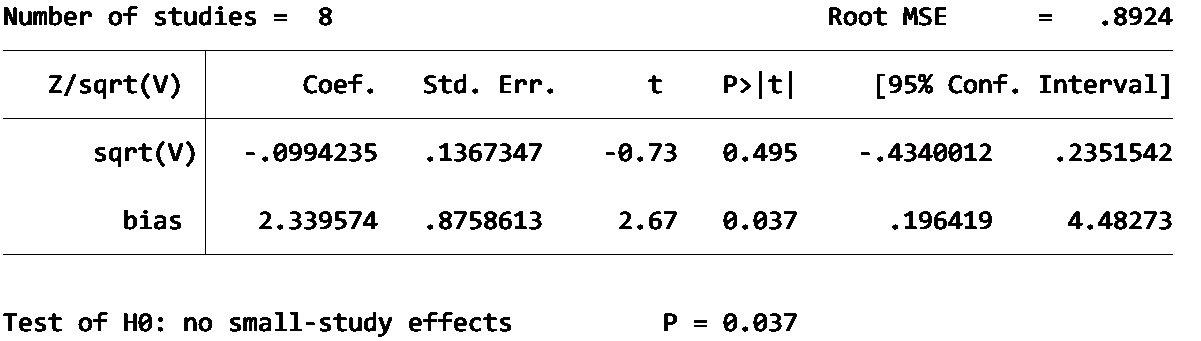

Supplement: Supplementary file 2 [file Image6.TIF]

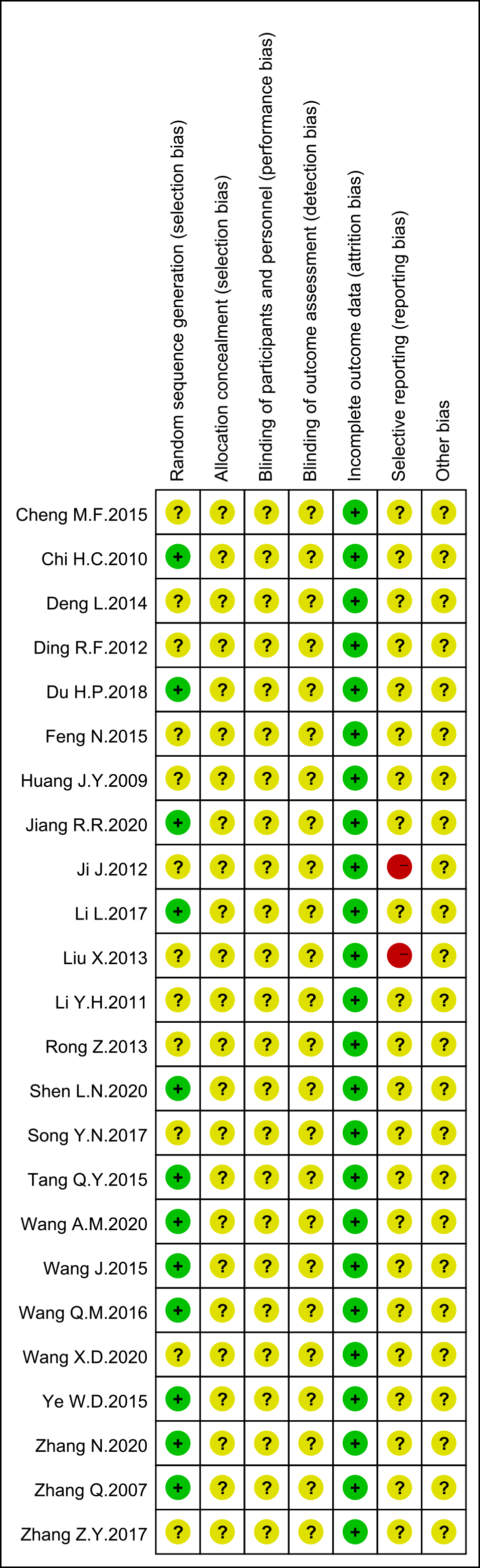

Supplement: Supplementary file 3 [file Image3.TIF]

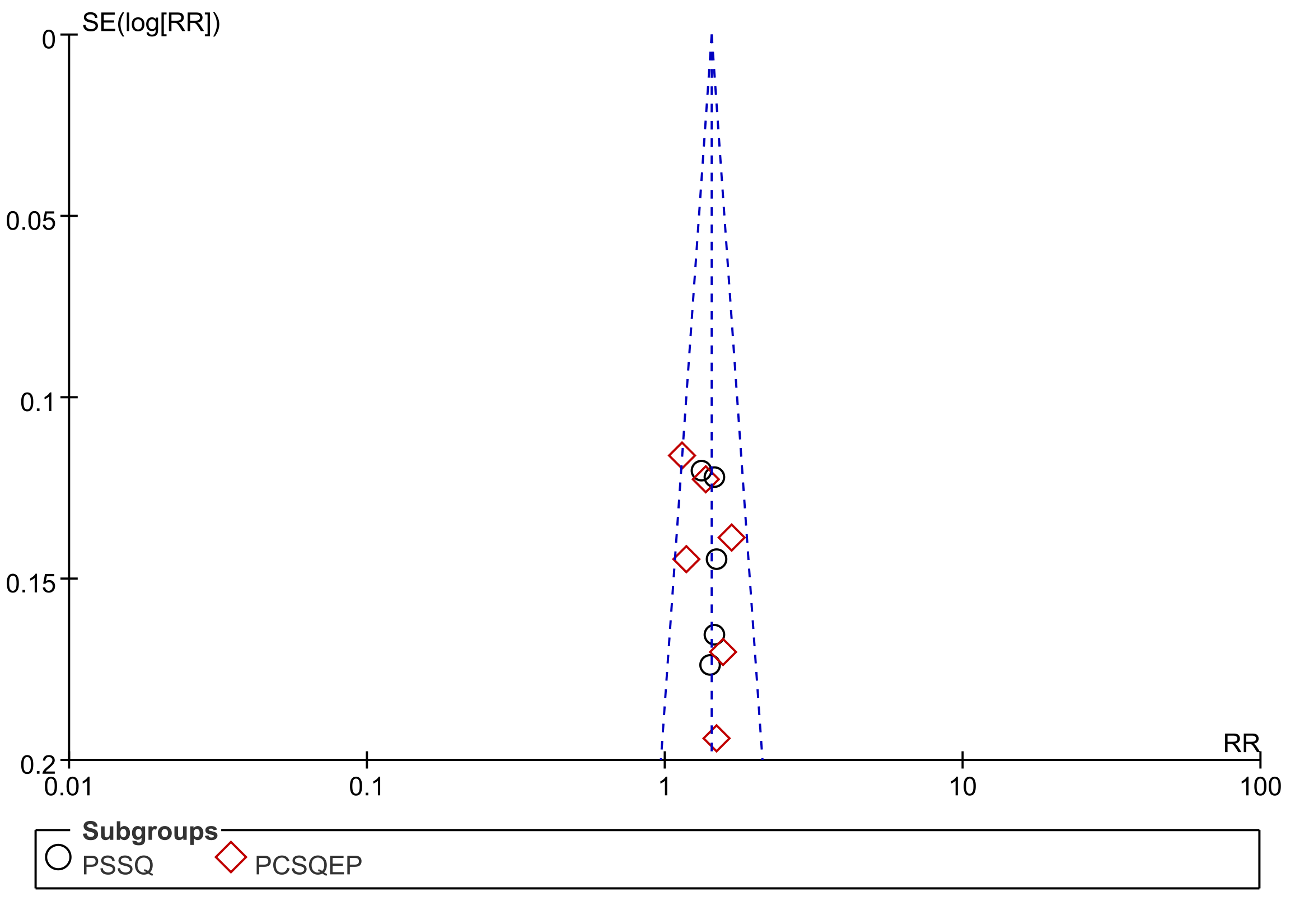

Supplement: Supplementary file 4 [file Image4.TIF]

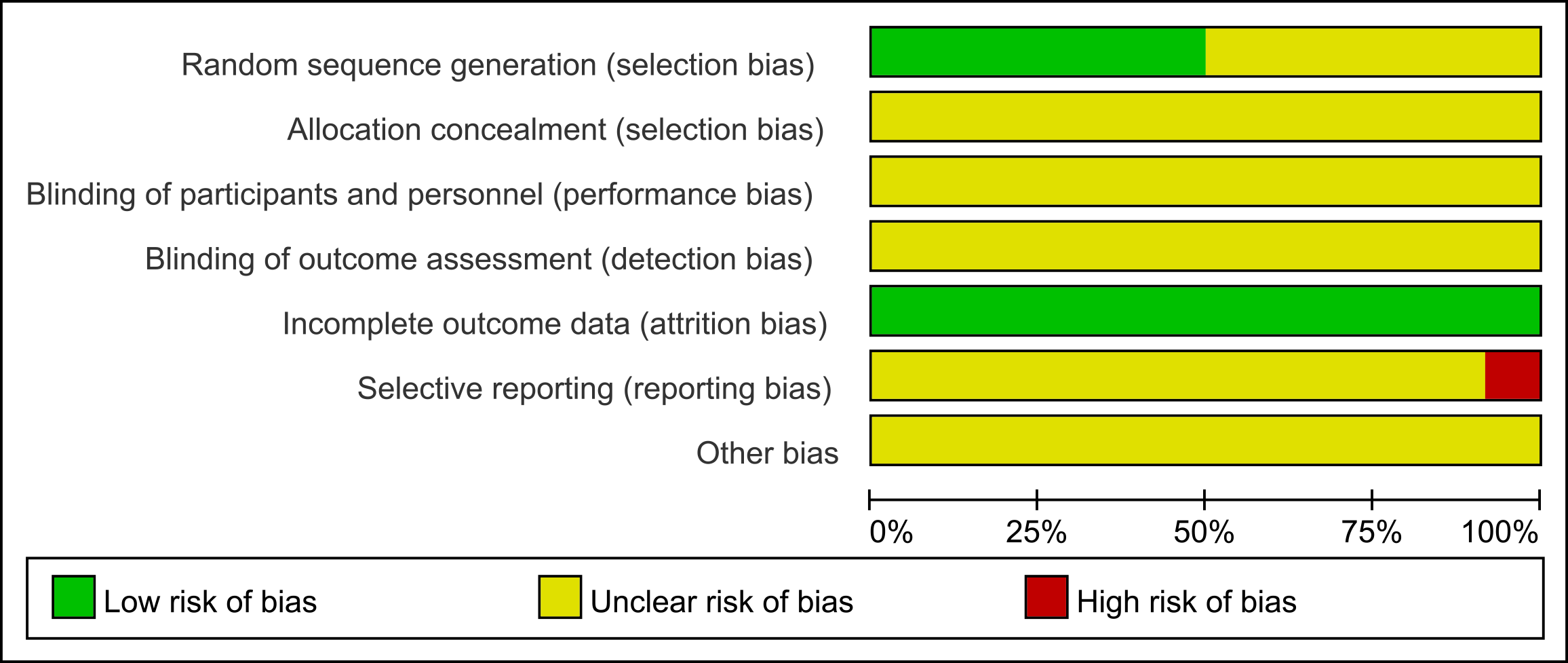

Supplement: Supplementary file 6 [file Image2.TIF]

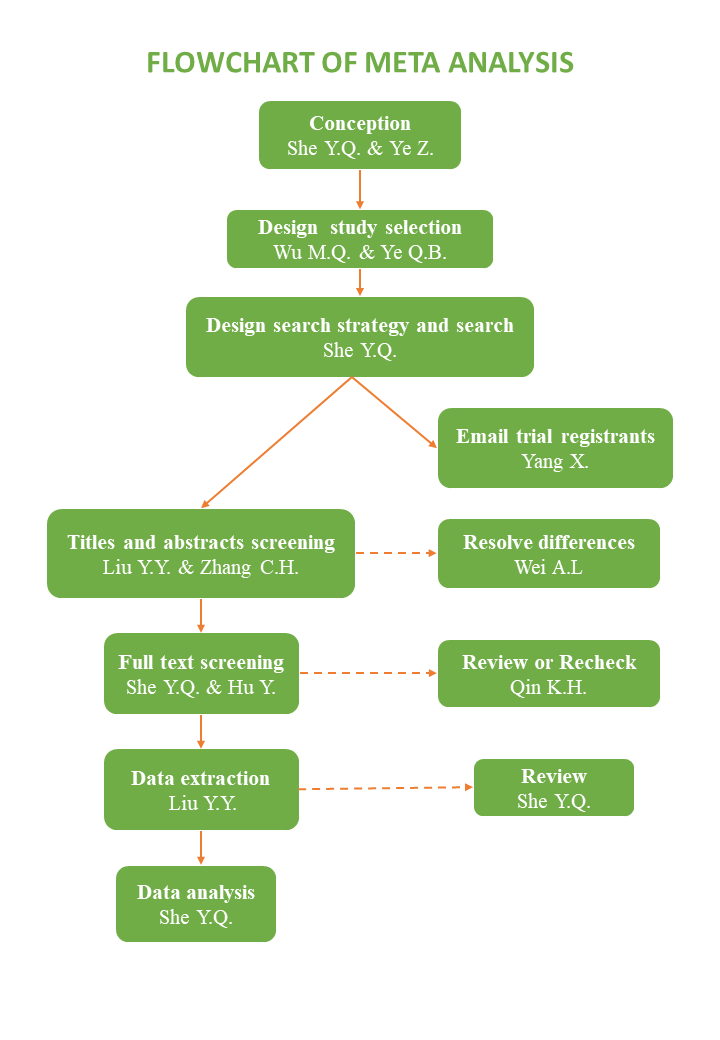

Supplement: Supplementary file 7 [file Image1.TIF]

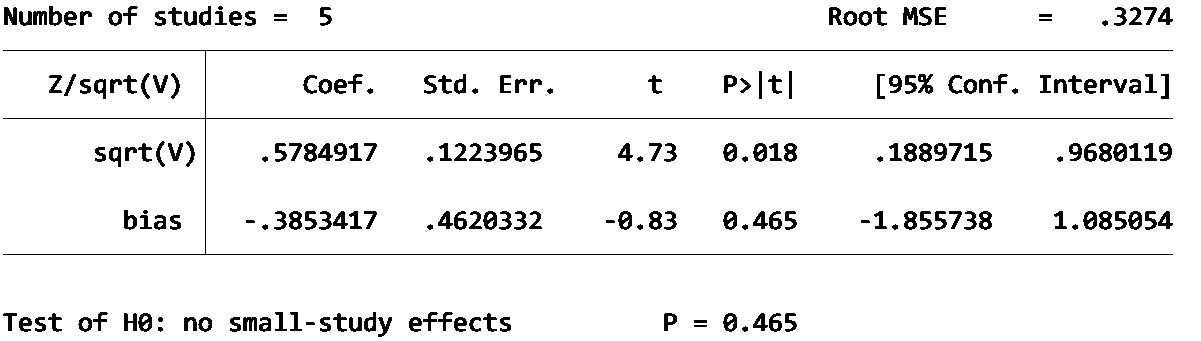

Supplement: Supplementary file 8 [file Image7.TIF]

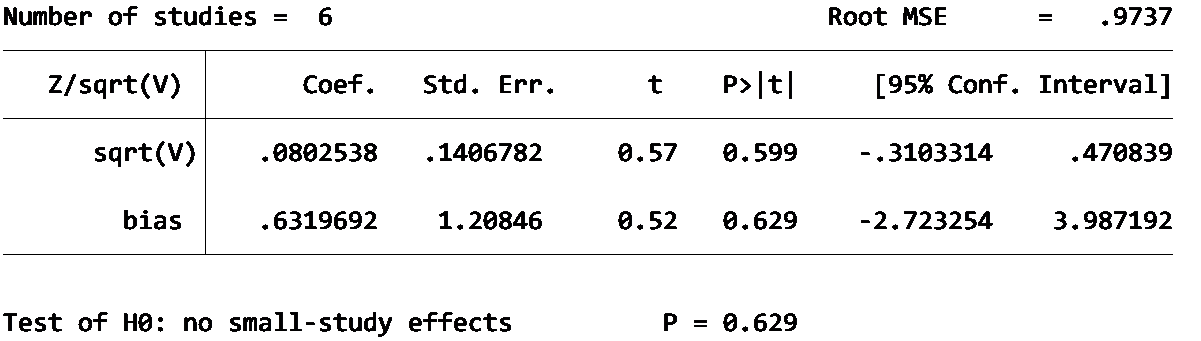

Supplement: Supplementary file 9 [file Image5.TIF]
