# Supplementary material for: The Therapeutic Principle of Combined Strengthening Qi and Eliminating Pathogens in Treating Middle-Advanced Primary Liver Cancer: A Systematic Review and Meta-Analysis
Source: Front Pharmacol. 2021 Oct 27;12:714287. doi: 10.3389/fphar.2021.714287 (PMC8578139; doi:10.3389/fphar.2021.714287)
Supplement: Supplementary file 5 [file DataSheet3.docx]

# Supplementary tables

## Supplementary table 1

The experimental design between the different qualities of RCTs

| ***The approach of RCTs design*** | ***High quality trials*** | ***Poor quality trials*** |
| --- | --- | --- |
| Setting | Describe the location, relevant dates and the detail of experimental process | Without the detail of executing processes |
| Participants information | Describe the general characteristic of patients and highlight the comparability of baseline | Without clinical information such as the experience of drug use or the comparability among groups |
| Inclusion criteria, exclusion criteria and diagnostic criteria | Expatiate the criteria of selection | Only present one of the three criteria-inclusion, exclusion and diagnostic |
| Therapy | Specify therapy for different groups | Present crude therapeutic schemes, such as non-dosage report |
| Variable and Therapeutic evaluation | Clarify all outcome and therapeutic evaluation | Without therapeutic evaluation |
| Statistical method | Describe all the statistical method | Some of the statistical methods do not report |

*The term of “Setting”, “Therapy” and “Statistical method” has been made a set of selection standards in the paragraph of “Type of studies”, “Types of intervention”, “Types of Comparison” and “Outcomes”. In the part of “Experimental design”, the term of “Participants information”, “Inclusion criteria, exclusion criteria and diagnostic criteria” and “Variable and Therapeutic evaluation” are the key point to elucidate.

## Supplementary table 2

The exclusive reasons based on PICOs criteria and study design during full-text screening

| ***Items*** | ***Exclusive reasons*** |
| --- | --- |
| Study design | Nonclinical trials (n=20)  Non-RCTs. (n=45) |
| Patients | Secondary liver cancer (n=13)  Without PLC stage (n=328)  Early PLC (n=120) |
| Intervention | Treatment without using Qi replenishing compound Chinese herbal medicine (n=129)  Chinese medicine without prescription (n=9)  Treatment duration less than one week or unknown (n=34) |
| Comparison | Without using TACE therapy or routine therapy after TACE (n=108) |
| Outcome | Irrelated outcome (n=11)  Insufficient data for statistical analysis (n=10) |
| Experimental design | Without efficacy evaluation standard (n=7)  Without diagnostic, inclusive or exclusive criteria (n=84)  Without clinical information or the comparability between two groups (n=3)  Without washout period (n=95)  Conference papers or dissertations (n=17) |

## Supplementary table 3

Details of interventions

| **First author and year** | **Components** |
| --- | --- |
| Zhang N.2020^△^ | *yù jīn* (the dried root tuber of *Curcuma aromatica* Salisb.) 30g, *bàn zhī lián* (the dried whole plant of *Scutellaria barbata* D.Don) 30g, *huáng qí* (the dried root of *Astragalus mongholicus* Bunge) 30g, *chén pí* (the dried riped peel of *Citrus × aurantium* L.) 15g, *jīn qián cǎo* (the dried whole plant of *Lysimachia christinae* Hance) 15g, *chái hú* (the dried root of Bupleurum chinense DC.) 10g, *mù xiāng* (the dried root of *Aucklandia costus Falc.*) 10g, *dǎng shēn* (the dried root of *Codonopsis pilosula* (Franch.) Nannf.) 12g, *jīn chán huā* (a fungus, the sporophore of *Isaia cicadae* Mique and the stroma of *Cordyceps cicadae* Shing). If jaundice appeared, *yīn chén* (the Aboveground Part of *Artemisia capillaris* Thunb.) was added; if ascites appeared, *long kuí* (the dried whole plant of *Solanum nigrum* L.); if nausea and vomiting appeared, *bàn xià* (the rhizome of *Pinellia ternata* (Thunb.) Makino), *jiāo shān zhā* (the processing ripe fruit of *Crataegus pinnatifida* Bunge), *jiāo shén qǔ* (the dried yeast preparations of wheat bran, flour, ricebean powder (*Vigna umbellata* (Thunb.) Ohwi and Ohashi), and bitter apricot seed powder (*Prunus mandshurica* (Maxim.) Koehne), *jiāo mài yá* (the processing ripe fruit of *Hordeum vulgare* L.), *xuán fù huā* (the flower head of *Inula japonica* Thunb.), *zhě shí* (Fe_2_O_3_) were added. |
| Shen L. N.2020^△^ | *chì sháo* (the dried root of *Paeonia lactiflora* Pall.) 10g, *líng zhī* (the dried sclerotium of *Ganoderma lucidum* (Leyss.exFr.) Karst.) 10g, *bái zhú* (the dried rhizome of *Atractylodes macrocephala* Koidz.) 10g, *wū yào* (the root tuber of *Lindera aggregata* (Sims) Kosterm.) 10g, *chuān xiōng* (the dried rhizome of *Conioselinum anthriscoides* 'Chuanxiong') 10g, *mǔ dān pí* (the root barks of *Paeonia × suffruticosa* Andrews) 10g, *chǎo zhǐ qiào* (the processing premature fruit of *Citrus × aurantium* L.) 10g, *táo rén* (the dried ripe seed of *Prunus persica* (L.) Batsch) 15g, *dāng guī* (the dried root of *Angelica sinensis* (Oliv.) Diels) 15g, *hóng huā* (the dried flower of *Carthamus tinctorius* L.) 15g, *zhì xiāng fù* (the processing rhizome of *Cyperus rotundus* L.) 6g, *yán hú suǒ* (the dried tuber of *Corydalis yanhusuo* (Y.H.Chou & Chun C.Hsu) W.T.Wang ex Z.Y.Su & C.Y.Wu) 6g, *gān cǎo* (the dried rhizome and root of *Glycyrrhiza glabra* L.) 5g. If liver pain appeared, *pú huáng* (the dried pollen of *Typha angustifolia* L.) 10g, *wǔ líng zhī* (the dried fences of *Trogopterus xanthipes* Milne-Ed-wards) 10g were added; if nausea and vomiting appeared, *huò xiāng* (the dried aboveground part of *Agastache rugosa* (Fisch. & C.A.Mey.) Kuntze) 10g, *zhú rú* (the dried caulis of *Bambusa tuldoides* Munro) 15g, *shì dì* (the dried persistent calyx of *Diospyros kaki* L.f.) 10g were added; if fever appeared, *bò hé* (the dried aboveground part of *Mentha canadensis* L.) 10g, *chái hú* (the dried root of Bupleurum chinense DC.) 10g were added; if malaise appeared, *huáng qí* (the dried root of *Astragalus mongholicus* Bunge) 15g, *xiān hè cǎo* (the dried aboveground part of *Agrimonia pilosa* Ledeb.) 10g, *shān zhū yú* (the dried ripe flesh of *Cornus officinalis* Siebold & Zucc.) 10g were added；if anorexia appeared, *shān zhā* (the dried ripe fruit of *Crataegus pinnatifida* Bunge) 10g, *chén pí* (the dried riped peel of *Citrus × aurantium* L.) 10g, *jī nèi jīn* (the dried inner wall of the stomach of *Gallus gallus domesticus* Brisson) 12g were added. |
| Feng N.2015^△^ | *huáng qí* (the dried root of *Astragalus mongholicus* Bunge) 14g, *dān shēn* (the dried root and rhizome of *Salvia miltiorrhiza* Bunge) 15g, *hóng huā* (the dried flower of *Carthamus tinctorius* L.) 6g, *táo rén* (the dried ripe seed of *Prunus persica* (L.) Batsch) 9g, *xiāng fù* (the dried rhizome of *Cyperus rotundus* L.) 10g, *mǔ dān pí* (the root barks of *Paeonia × suffruticosa* Andrews) 12g, *bái huā shé shé cǎo* (the dried whole plant of *Scleromitrion diffusum* (Willd.) R.J.Wang) 16g, *zhǐ qiào* (the dried premature fruit of *Citrus × aurantium* L.) 12g, *xuán shēn* (the dried root of *Scrophularia ningpoensis* Hemsl.) 4g. |
| Cheng M. F.2015^△^ | *huáng qí* (the dried root of *Astragalus mongholicus* Bunge) 20g, *lián qiào* (the dried ripe fruit of *Forsythia suspensa* (Thunb.) Vahl) 20g, *dāng guī*(the dried root of *Angelica sinensis* (Oliv.) Diels) 15g, *tài zǐ shēn* (the dried toot tuber of *Pseudostellaria heterophylla* (Miq.) Pax) 15g, *chén pí* (the dried riped peel of *Citrus × aurantium* L.) 20g, *huáng jīng* (the dried rhizome of *Polygonatum sibiricum* Redouté) 15g, *shān zhū yú* (the dried ripe flesh of *Cornus officinalis* Siebold & Zucc.) 30g, *nǚ zhēn zǐ* (the dried ripe fruit of *Ligustrum lucidum* W.T.Aiton) 15g, *fú líng* (a fungus, the dried sclerotium of *Poria cocos* (Schw.) Wolf) 18g, *shēng shān zhā* (the dried ripe fruit of *Crataegus pinnatifida* Bunge) 25g, *shú zhū gān* (the cooked liver of *Sus scrofa domestica* Brisson) 25g, *dān shēn* (the dried root and rhizome of *Salvia miltiorrhiza* Bunge) 10g, *sān qī fěn* (the dried root and rhizome of *Panax notoginseng* (Burkill) F.H.Chen) 5g. If qi deficiency appeared, *bái zhú* (the dried rhizome of *Atractylodes macrocephala* Koidz.), *yì yǐ rén* (the dried seed of *Coix lacryma-jobi var. ma-yuen* (Rom.Caill.) Stapf) were added; if diarrhea appeared, *huáng lián* (the dried rhizome of *Coptis chinensis* Franch.), *ròu guì* (the dried bark of *Neolitsea cassia* (L.) Kosterm.) were added. |
| Deng L.2014^△^ | *dǎng shēn* (the dried root of *Codonopsis pilosula* (Franch.) Nannf.) 15g, *bái zhú* (the dried rhizome of *Atractylodes macrocephala* Koidz.) 15g, *fú líng* (a fungus, the dried sclerotium of *Poria cocos* (Schw.) Wolf) 15g, *huáng qí* (the dried root of Astragalus mongholicus Bunge) 30g, *chái hú* (the dried root of *Bupleurum chinense* DC.) 10g, *bái sháo* (the dried root of *Paeonia lactiflora* Pall.) 15g, *dān shēn* (the dried root and rhizome of *Salvia miltiorrhiza* Bunge) 15g, *jī nèi jīn* (the dried inner wall of the stomach of *Gallus gallus domesticus* Brisson) 8g, *nǚ zhēn zǐ* (the dried ripe fruit of *Ligustrum lucidum* W.T.Aiton) 10g, *shí jiàn chuān* (the dried whole plant of *Salvia chinese* Benth) 15g, *gān cǎo* (the dried rhizome and root of *Glycyrrhiza glabra* L.) 6g. If abdominal pain appeared, *chuān liàn zǐ* (the dried ripe fruit of *Melia azedarach* L.) 15g, *yù jīn* (the dried root tuber of *Curcuma aromatica* Salisb.) 15g were added. *yuán hú* (the dried tuber of *Corydalis yanhusuo* (Y.H.Chou & Chun C.Hsu) W.T.Wang ex Z.Y.Su & C.Y.Wu) 15g; if fever appeared, *shí gāo* (CaSO_4_ • 2H_2_O) 30g, *hán shuǐ shí* (CaSO_4_ • 2H_2_O, CaCO_3_) 30g were added. The dose of *chái hú* (the dried root of *Bupleurum chinense* DC.) was changed to 15g. If jaundice appeared, *yīn chén* (the Aboveground Part of *Artemisia capillaris* Thunb.) 30g, *dà huáng* (the dried root and rhizome of Rheum palmatum L.) 8-12g, *zhī zǐ* (the dried ripe fruit of *Gardenia jasminoides* J.Ellis) 10g were added；if nausea and vomiting appeared, *bàn xià* (the rhizome of *Pinellia ternata* (Thunb.) Makino) 15g, *chén pí* (the dried riped peel of *Citrus × aurantium* L.) 15g, *zhě shí* (Fe_2_O_3_) 30g were added; if abdominal distension appeared, *dà fù pí* (the dried peel of *Areca catechu* L.) 30g, *zhū líng* (a fungus, the dried sclerotium of *Polyporus umbellatus* (Pers.) Fries) 15g were appeared. |
| Liu X.2013^△^ | *chái hú* (the dried root of *Bupleurum chinense* DC.), *huáng qín* (the dried root of *Scutellaria baicalensis* Georgi), *chǎo bái zhú* (the processing rhizome of *Atractylodes macrocephala* Koidz.), *yù jīn* (the dried root tuber of *Curcuma aromatica* Salisb.), *zhǐ qiào* (the dried premature fruit of *Citrus × aurantium* L.), *fú líng* (a fungus, the dried sclerotium of *Poria cocos* (Schw.) Wolf), *fú shén* (a fungus, the dried sclerotium with host pine root of *Poria cocos* (Schw.) Wolf), *chì sháo* (the dried root of *Paeonia lactiflora* Pall.), *bái sháo* (the dried root of *Paeonia lactiflora* Pall.), *chén pí* (the dried riped peel of *Citrus × aurantium* L.), *chǎo dāng guī* (the processing root of *Angelica sinensis* (Oliv.) Diels). If abdominal distension appeared, *zǐ sū gěng* (the dried stem of *Perilla frutescens* (L.) Britton), *hòu pò* (the dried bark of *Magnolia officinalis* Rehder & E.H.Wilson) were added; If nausea and vomiting appeared, *shēng jiāng* (the flesh rhizome of *Zingiber officinale* Roscoe), *zhú rú* (the dried caulis of *Bambusa tuldoides* Munro), *jiāng bàn xià* (the processing rhizome of *Pinellia ternata* (Thunb.) Makino) were added; if abdominal pain appeared, *wū yào* (the root tuber of *Lindera aggregata* (Sims) Kosterm.), *yán hú suǒ* (the dried tuber of *Corydalis yanhusuo* (Y.H.Chou & Chun C.Hsu) W.T.Wang ex Z.Y.Su & C.Y.Wu) were added; if ascites appeared, *dà fù pí* (the dried peel of *Areca catechu* L.), *zhū líng* (a fungus, the dried sclerotium of *Polyporus umbellatus* (Pers.) Fries), *zé xiè* (the dried tuber of Alisma *plantago-aquatica subsp. orientale* (Sam.) Sam.) were added; if jaundice appeared, *yīn chén* (the Aboveground Part of *Artemisia capillaris* Thunb.) was added. |
| Ji J.2012^△^ | *chái hú* (the dried root of *Bupleurum chinense* DC.) 15g, *dāng guī* (the dried root of *Angelica sinensis* (Oliv.) Diels) 15g, *fú líng* (a fungus, the dried sclerotium of *Poria cocos* (Schw.) Wolf) 10g, *bái sháo* (the dried root of *Paeonia lactiflora* Pall.) 10g, *bái zhú* (the dried rhizome of *Atractylodes macrocephala* Koidz.) 10g, *gān cǎo* (the dried rhizome and root of *Glycyrrhiza glabra* L.) 9g, *wēi jiāng* (the processing rhizome of *Zingiber officinale* Roscoe) 9g, *bò hé* (the dried aboveground part of *Mentha canadensis* L.) 9g, *bái huā shé shé cǎo* (the dried whole plant of *Scleromitrion diffusum* (Willd.) R.J.Wang) 15g, *tài zǐ shēn* (the dried toot tuber of *Pseudostellaria heterophylla* (Miq.) Pax) 30g, *zhǐ qiào* (the dried premature fruit of *Citrus × aurantium* L.) 10g. |
| Li Y. H.2011^△^ | *chái hú* (the dried root of *Bupleurum chinense* DC.) 10g, *yù jīn* (the dried root tuber of *Curcuma aromatica* Salisb.) 10g, *chuān liàn zǐ* (the dried ripe fruit of *Melia azedarach* L.) 10g, *dāng guī* (the dried root of *Angelica sinensis* (Oliv.) Diels) 10g, *chì sháo* (the dried root of *Paeonia lactiflora* Pall.) 10g, *mǔ* *dān pí* (the root barks of *Paeonia × suffruticosa* Andrews) 10g, *rén shēn* (the dried rhizome and root of *Panax ginseng* C.A.Mey.) 10g, *fú líng* (a fungus, the dried sclerotium of *Poria cocos* (Schw.) Wolf) 10g, *bái zhú* (the dried rhizome of *Atractylodes macrocephala* Koidz.) 10g, *yì yǐ rén* (the dried seed of *Coix lacryma-jobi var. ma-yuen* (Rom.Caill.) Stapf) 30g, *mù xiāng* (the dried root of *Aucklandia costus Falc.*) 10g, *fǎ bàn xià* (the processing rhizome of *Pinellia ternata* (Thunb.) Makino) 10g, *shā rén* (the dried ripe fruit of *Wurfbainia villosa* (Lour.) Skornick. & A.D.Poulsen) 5g, *jī nèi jīn* (the dried inner wall of the stomach of *Gallus gallus domesticus* Brisson) 5g, *shān zhā* (the dried ripe fruit of *Crataegus pinnatifida* Bunge) 10g, *nǚ zhēn zǐ* (the dried ripe fruit of *Ligustrum lucidum* W.T.Aiton) 10g, *tú sī zǐ* (the dried ripe seed of *Cuscuta chinensis* Lam.) 10g, *mò hàn lián* (the dried aboveground part of *Eclipta prostrata* (L.) L.) 10g, *bǔ gǔ zhī* (the dried ripe fruit of *Cullen corylifolium* (L.) Medik.) 10g, *dì huáng* (the root tuber of *Rehmannia glutinosa* (Gaertn.) DC.) 10g, *gǒu qǐ zǐ* (the dried ripe fruit of *Lycium barbarum* L.) 15g. |
| Chi H. C.2010^△^ | *chái hú* (the dried root of *Bupleurum chinense* DC.) 10g, *huáng qín* (the dried root of *Scutellaria baicalensis* Georgi) 10g, *dǎng shēn* (the dried root of *Codonopsis pilosula* (Franch.) Nannf.) 10g, *bái zhú* (the dried rhizome of *Atractylodes macrocephala* Koidz.) 10g, *dāng guī* (the dried root of *Angelica sinensis* (Oliv.) Diels) 10g, *chén pí* (the dried riped peel of *Citrus × aurantium* L.) 10g, *chuān liàn zǐ* (the dried ripe fruit of *Melia azedarach* L.) 10g, *yán hú suǒ* (the dried tuber of *Corydalis yanhusuo* (Y.H.Chou & Chun C.Hsu) W.T.Wang ex Z.Y.Su & C.Y.Wu) 20g, *zhì gān cǎo* (the processing rhizome and root of *Glycyrrhiza glabra* L.) 6g.If chest discomfort appeared, *xiāng fù* (the dried rhizome of *Cyperus rotundus* L.), *bàn xià* (the rhizome of *Pinellia ternata* (Thunb.) Makino) were added; if liver pain appeared, *jiàng xiāng* (the dried stem and root of *Dalbergia odorifera* T.C.Chen), *chì sháo* (the dried root of *Paeonia lactiflora* Pall.) were added; if constipation appeared, *dà huáng* (the dried root and rhizome of Rheum palmatum L.), *hòu pò* (the dried bark of *Magnolia officinalis* Rehder & E.H.Wilson) were added；if abdominal distension appeared, *bái biǎn dòu* (the dried ripe seed of *Lablab purpureus subsp.* purpureus), *zhǐ qiào* (the dried premature fruit of *Citrus × aurantium* L.) were added; if nausea and vomiting appeared, *zhú rú*(the dried caulis of *Bambusa tuldoides* Munro), *jiāng bàn xià* (the processing rhizome of *Pinellia ternata* (Thunb.) Makino) were added; if hepatic dysfunction appeared, *wǔ wèi zǐ* (the dried ripe fruit of *Schisandra chinensis* (Turcz.) Baill.), *jiāng huáng* (the dried rhizome of *Curcuma longa* L.), *yīn chén* (the Aboveground Part of *Artemisia capillaris* Thunb.) were added. |
| Wang A. M.2020^△^ | *rén shēn* (the dried rhizome and root of *Panax ginseng* C.A.Mey.) 30g, *fú líng* (a fungus, the dried sclerotium of Poria cocos (Schw.) Wolf) 30g, *bái zhú* (the dried rhizome of *Atractylodes macrocephala* Koidz.) 30g, *dāng guī* (the dried root of *Angelica sinensis* (Oliv.) Diels) 30g, *chuān xiōng* (the dried rhizome of *Conioselinum anthriscoides* 'Chuanxiong') 30g, *bái sháo* (the dried root of *Paeonia lactiflora* Pall.) 30g, *shú dì huáng* (the processing root tuber of *Rehmannia glutinosa* (Gaertn.) DC.) 30g, *zhì gān cǎo* (the processing rhizome and root of *Glycyrrhiza glabra* L.) 30g, *shēng jiāng* (the flesh rhizome of *Zingiber officinale* Roscoe) 3 slice, *dà zǎo* (the dried ripe fruit of *Ziziphus jujuba* Mill.) 5 piece. If pathogenic heat appeared, *huáng qín* (the dried root of *Scutellaria baicalensis* Georgi), *shí gāo* (CaSO4 • 2H2O), *zhī mǔ* (the dried rhizome of *Anemarrhena asphodeloides* Bunge) were added; if pain appeared, *yán hú suǒ* (the dried tuber of *Corydalis yanhusuo* (Y.H.Chou & Chun C.Hsu) W.T.Wang ex Z.Y.Su & C.Y.Wu), *bái sháo* (the dried root of *Paeonia lactiflora* Pall.) were added; if nausea and vomiting appeared, *jiāng bàn xià* (the processing rhizome of *Pinellia ternata* (Thunb.) Makino), *zhú rú* (the dried caulis of *Bambusa tuldoides* Munro) were added；if dampness-heat appeared, *yīn chén* (the Aboveground Part of *Artemisia capillaris* Thunb.), *hǔ zhàng* (the dried rhizome and root of *Reynoutria japonica* Houtt.) were added; if blood stasis appeared, *dān shēn* (the dried root and rhizome of *Salvia miltiorrhiza* Bunge), *biē jiǎ* (the carapace of *Trionyx sinensis* Wiegmann) were added; if yin deficiency, *dì huáng* (the root tuber of *Rehmannia glutinosa* (Gaertn.) DC.), *shā shēn* (the dried root of *Adenophora triphylla* (Thunb.) A.DC.) were added; if qi stagnation appeared, *chén pí* (the dried riped peel of *Citrus × aurantium* L.), *fó shǒu* (the dried fruit of *Citrus medica* L.) were added. |
| Song Y. N.2017^△^ | *ròu cōng róng* (the fleshy stem of *Cistanche deserticola* Ma) 30g, *huáng qí* (the dried root of *Astragalus mongholicus* Bunge) 30g, *dì huáng* (the root tuber of *Rehmannia glutinosa* (Gaertn.) DC.) 15g, *huáng qín* (the dried root of *Scutellaria baicalensis* Georgi) 15g, *bàn zhī lián* (the dried whole plant of *Scutellaria barbata* D.Don) 30g. If pathogenic heat appeared, *yīn chén* (the Aboveground Part of *Artemisia capillaris* Thunb.), *zhī zǐ* (the dried ripe fruit of *Gardenia jasminoides* J.Ellis); if nausea and vomiting appeared, *fǎ bàn xià* (the processing rhizome of *Pinellia ternata* (Thunb.) Makino), *shēng jiāng* (the flesh rhizome of *Zingiber officinale* Roscoe), *xuán fù huā* (the flower head of *Inula japonica* Thunb.) were added; if pain appeared, *màn jīng zǐ* (the dried ripe fruit of *Vitex trifolia* L.), *sān qī* (the dried root and rhizome of *Panax notoginseng* (Burkill) F.H.Chen), *yán hú suǒ* (the dried tuber of *Corydalis yanhusuo* (Y.H.Chou & Chun C.Hsu) W.T.Wang ex Z.Y.Su & C.Y.Wu), *mù xiāng* (the dried root of *Aucklandia costus Falc.*), *yù jīn* (the dried root tuber of *Curcuma aromatica* Salisb.) were added. |
| Ye W. D.2015^△^ | *zhì huáng qí* (the processing root of *Astragalus mongholicus* Bunge), *chǎo dǎng shēn* (the processing root of *Codonopsis pilosula* (Franch.) Nannf.), *chǎo fú líng* (a fungus, the processing sclerotium of Poria cocos (Schw.) Wolf), *chǎo yì yǐ rén* (the processing seed of *Coix lacryma-jobi var. ma-yuen* (Rom.Caill.) Stapf), *chǎo bái zhú* (the processing rhizome of *Atractylodes macrocephala* Koidz.), *zhì gān cǎo* (the processing rhizome and root of *Glycyrrhiza glabra* L.), *dān shēn* (the dried root and rhizome of *Salvia miltiorrhiza* Bunge), *yù jīn* (the dried root tuber of *Curcuma aromatica* Salisb.), *zhǐ qiào* (the dried premature fruit of *Citrus × aurantium* L.), *chǎo bái sháo* (the processing root of *Paeonia lactiflora* Pall.). If liver and kidney deficiency appeared, *dāng guī* (the dried root of *Angelica sinensis* (Oliv.) Diels), *shān zhū yú* (the dried ripe flesh of *Cornus officinalis* Siebold & Zucc.), *gǒu qǐ zǐ* (the dried ripe fruit of *Lycium barbarum* L.) were added； if ascites appeared, *zhū líng* (a fungus, the dried sclerotium of *Polyporus umbellatus* (Pers.) Fries), *zé lán* (the dried aboveground part of *Lycopus lucidus var. hirtus* (Regel) Makino & Nemoto); if pain and blood stasis appeared, *wǔ líng zhī* (the dried fences of *Trogopterus xanthipes* Milne-Ed-wards), *pú huáng* (the dried pollen of *Typha angustifolia* L.) were added; if jaundice appeared, *yīn chén* (the Aboveground Part of *Artemisia capillaris* Thunb.) was added; if yin deficiency, *shā shēn* (the dried root of *Adenophora triphylla* (Thunb.) A.DC.), *shí hú* (the stem of *Dendrobium nobile* Lindl.) were added; if hemorrhage appeared, *bái máo gēn* (the dried rhizome of *Imperata cylindrica* (L.) P.Beauv.), *xiān hè cǎo* (the dried aboveground part of *Agrimonia pilosa* Ledeb.) were added; if fever appeared, *dì gǔ pí* (the dried root bark of *Lycium barbarum* L.), *qīng hāo* (the dried aboveground part of *Artemisia annua* L.), *mǔ* *dān pí* (the root barks of *Paeonia × suffruticosa* Andrews) were added. |
| Huang J. Y.2009^#^ | *rén shēn* (the dried rhizome and root of *Panax ginseng* C.A.Mey.), *jiāng cán* (the dried insect body of *Bombyx mori* Linnaeus), *dǎn shēn* (the dried root and rhizome of *Salvia miltiorrhiza* Bunge), *huáng qí* (the dried root of *Astragalus mongholicus* Bunge), *dāng guī* (the dried root of *Angelica sinensis* (Oliv.) Diels), b*īng piàn*(Borneol), *é zhú* (the dried rhizoma of *Curcuma kwangsiensis* S.G.Lee & C.F.Liang), *shān cí gū* (the dried pseudobulb of *Cremastra appendiculata* (D.Don) Makino), *mă qián zĭ* (the mature seed of *Strychnos nux-vomica* L.), *fēng fáng* (the nest of *Polistes　olivaceous* (DeGeer)), *yā dăn zĭ* (the mature fruit of *Brucea javanica* (L.) Merr.), *rén gōng niú huáng* (*Calculus Bovis Artifactus*). |
| Wang J.2015^#^ | *huáng qí* (the dried root of *Astragalus mongholicus* Bunge), *nǚ zhēn zǐ* (the dried ripe fruit of *Ligustrum lucidum* W.T.Aiton), *rén shēn* (the dried rhizome and root of *Panax ginseng* C.A.Mey.), *líng zhī* (the dried sclerotium of *Ganoderma lucidum* (Leyss.exFr.) Karst.), *jiǎo gǔ lán* (the dried whole plant of *Gynostemma pentaphyllum* (Thunb.) Makin), *chǎo bái zhú* (the processing rhizome of *Atractylodes macrocephala* Koidz.), *bàn zhī lián* (the dried whole plant of *Scutellaria barbata* D.Don), *bái huā shé shé cǎo* (the dried whole plant of *Scleromitrion diffusum* (Willd.) R.J.Wang), *fú líng* (a fungus, the dried sclerotium of Poria cocos (Schw.) Wolf), *jī nèi jīn* (the dried inner wall of the stomach of *Gallus gallus domesticus* Brisson)、*shé méi* (the dried whole plant of *Potentilla indica* (Andrews) Th.Wolf), *bái yīng* (the dried plant or root of *Solanum lyratum* Thunb.), *yīn chén* (the Aboveground Part of *Artemisia capillaris* Thunb.), *xú cháng qīng* (the dried root and rhizome of Vincetoxicum mukdenense Kitag.), *é zhú* (the dried rhizoma of *Curcuma kwangsiensis* S.G.Lee & C.F.Liang), *tŭ biē chóng* (the dried insect body of *Eupolyphaga sinensis* Walker) |
| Rong Z.2013^#^ | *rén shēn* (the dried rhizome and root of *Panax ginseng* C.A.Mey.), *bái huā shé shé cǎo* (the dried whole plant of *Scleromitrion diffusum* (Willd.) R.J.Wang), *wú gōng* (the dried insect body of *Scolopendra subspinipes mutilans* L. Koch), *bā jiăo lián* (the dried rhizoma and root of *Podophyllum versipelle* Hance), *tŭ biē chóng* (the dried insect body of *Eupolyphaga sinensis* Walker). |
| Wang Q. M.2016^#^ | *dǎng shēn* (the dried root of *Codonopsis pilosula* (Franch.) Nannf.) 10g, *bái zhú* (the dried rhizome of *Atractylodes macrocephala* Koidz.) 10g, *huáng qí* (the dried root of *Astragalus mongholicus* Bunge) 20g, *fú líng* (a fungus, the dried sclerotium of Poria cocos (Schw.) Wolf) 10g, *chái hú* (the dried root of *Bupleurum chinense* DC.) 10g, *xiāng fù* (the dried rhizome of *Cyperus rotundus* L.) 10g, *chén pí* (the dried riped peel of *Citrus × aurantium* L.) 10g, *cù biē jiǎ* (the processing carapace of *Trionyx sinensis* Wiegmann) 15g, *táo rén* (the dried ripe seed of *Prunus persica* (L.) Batsch) 10g, *dān shēn* (the dried root and rhizome of *Salvia miltiorrhiza* Bunge) 15g, *shēng mǔ lì* (the shell of *Ostrea gigas* Thunberg) 30g, *bàn zhī lián* (the dried whole plant of *Scutellaria barbata* D.Don) 30g, *ái huā shé shé cǎo* (the dried whole plant of *Scleromitrion diffusum* (Willd.) R.J.Wang) 30g, *gān cǎo* (the dried rhizome and root of *Glycyrrhiza glabra* L.) 5g, *zăo xiū* (the dried rhizome of *Paris polyphylla var. yunnanensis* (Franch.) Hand. -Mazz.) 9g. |
| Du H. P.2018^#^ | *biē jiǎ jiāo* (Colla Carapacis Trionycis), *ē jiāo* (Colla Corii Asini), *chái hú* (the dried root of *Bupleurum chinense* DC.), *huáng qín* (the dried root of *Scutellaria baicalensis* Georgi), *zhì bàn xià* (the processing rhizome of *Pinellia ternata* (Thunb.) Makino), *dǎng shēn* (the dried root of *Codonopsis pilosula* (Franch.) Nannf.), *gān jiāng* (the dried *Zingiber officinale* Roscoe), *jiāng hòu pò* (the processing bark of *Magnolia officinalis* Rehder & E.H.Wilson), *guì zhī* (the dried bark of *Cinnamomum cassia* (L.) J.Presl), *chǎo bái sháo* (the processing root of *Paeonia lactiflora* Pall.), *shè gān* (the dried rhizome of *Iris domestica* (L.) Goldblatt & Mabb.), *táo rén* (the dried ripe seed of *Prunus persica* (L.) Batsch), *mǔ dān pí* (the root barks of *Paeonia × suffruticosa* Andrews), *dà huáng* (the dried root and rhizome of Rheum palmatum L.), *líng xiāo huā* (the dried flower of *Campsis grandiflora* (Thunb.) K.Schum.), *tíng lì zǐ* (the dried ripe seed of *Descurainia sophia* (L.) Webb ex Prantl), *shí wěi* (the dried leaves of *Pyrrosia lingua* (Thunb.) Farw.), *qú mài* (the dried aboveground part of *Dianthus chinensis* L.).*tŭ biē chóng* (the dried insect body of *Eupolyphaga sinensis* Walker), *fēng fang* (the nest of *Polistes　olivaceous* (DeGeer)),*qiāng lǎng* (the dried insect body of *Catharsius molossus* (Linnaeus)),*shǔ fù* (the dried insect body of *Armadillidium vurgare* (Latrelle)), *xiāo shí* (Saltpetre). |
| Wang X. D.2020^#^ | *huáng qí* (the dried root of *Astragalus mongholicus* Bunge) 30g, *bái zhú* (the dried rhizome of *Atractylodes macrocephala* Koidz.) 15g, *nǚ zhēn zǐ* (the dried ripe fruit of *Ligustrum lucidum* W.T.Aiton) 15g, *gǒu qǐ zǐ* (the dried ripe fruit of *Lycium barbarum* L.) 15g, *dān shēn* (the dried root and rhizome of *Salvia miltiorrhiza* Bunge), *xiāng fù* (the dried rhizome of *Cyperus rotundus* L.) 10g, *bàn zhī lián* (the dried whole plant of *Scutellaria barbata* D.Don) 15g, *xià kū cǎo* (the dried fruit spike of *Prunella vulgaris* L.) 15g, *tŭ biē chóng* (the dried insect body of *Eupolyphaga sinensis* Walker) 15g, *é zhú* (the dried rhizoma of *Curcuma kwangsiensis* S.G.Lee & C.F.Liang) 15g.If qi deficiency appeared, *tài zǐ shēn* (the dried toot tuber of *Pseudostellaria heterophylla* (Miq.) Pax) 30g, *shān yào* (the dried rhizome of *Dioscorea oppositifolia* L.) 15g, *fú líng* (a fungus, the dried sclerotium of Poria cocos (Schw.) Wolf) 15g were added. if yin deficiency appeared, *běi shā shēn* (the dried root of *Glehnia littoralis* (A.Gray) F.Schmidt ex Miq.) 15g, *mài dōng* (the dried root tuber of *Ophiopogon japonicus* (Thunb.) Ker Gawl.) 15g, *dì huáng* (the root tuber of *Rehmannia glutinosa* (Gaertn.) DC.) 15g were added; if blood stasis appeared, *yù jīn* (the dried root tuber of *Curcuma aromatica* Salisb.) 15g, *shí jiàn chuān* (the dried whole plant of *Salvia chinese* Benth) 15g, *táo rén* (the dried ripe seed of *Prunus persica* (L.) Batsch) 10g were added; if pathogenic heat appeared, *chóng lóu* (the dried rhizome of *Paris polyphylla var. yunnanensis* (Franch.) Hand.-Mazz.) 15g, *hǔ zhàng* (the dried rhizome and root of *Reynoutria japonica* Houtt.) 15g, *bái huā shé shé cǎo* (the dried whole plant of *Scleromitrion diffusum* (Willd.) R.J.Wang) 15g, *pú gōng yīng* (the dried whole plant of *Taraxacum mongolicum* Hand.-Mazz.) 15g were added. |
| Jiang R. R.2020^#^ | *rén shēn* (the dried rhizome and root of *Panax ginseng* C.A.Mey.) 10g, *chái hú* (the dried root of *Bupleurum chinense* DC.) 12g, *xiāng fù* (the dried rhizome of *Cyperus rotundus* L.) 12g, *yīn chén* (the Aboveground Part of *Artemisia capillaris* Thunb.) 30g, *jiāng bàn xià* (the processing rhizome of *Pinellia ternata* (Thunb.) Makino) 12g, 竹茹(the dried caulis of *Bambusa tuldoides* Munro) *bái huā shé shé cǎo* (the dried whole plant of *Scleromitrion diffusum* (Willd.) R.J.Wang) 30g, *bái zhú* (the dried rhizome of *Atractylodes macrocephala* Koidz.) 15g, *zhǐ qiào* (the dried immature fruit of *Citrus × aurantium* L.) 10g, *fú líng* (a fungus, the dried sclerotium of Poria cocos (Schw.) Wolf) 15g, *dāng guī* (the dried root of *Angelica sinensis* (Oliv.) Diels) 12g, *bái sháo* (the dried root of *Paeonia lactiflora* Pall.) 12g, *biē jiǎ* (the carapace of *Trionyx sinensis* Wiegmann) 24g, *sān qī* (the dried root and rhizome of *Panax notoginseng* (Burkill) F.H.Chen) 10g, *niú xī* (the dried root of *Achyranthes bidentata* Blume) 30g, *shēng jiāng* (the flesh rhizome of *Zingiber officinale* Roscoe) 10g, *gān cǎo* (the dried rhizome and root of *Glycyrrhiza glabra* L.) 6g, *é zhú* (the dried rhizoma of *Curcuma kwangsiensis* S.G.Lee & C.F.Liang) 12g. |
| Tang Q. Y.2015^#^ | *bái huā shé shé cǎo* (the dried whole plant of *Scleromitrion diffusum* (Willd.) R.J.Wang) 20g, *bàn zhī lián* (the dried whole plant of *Scutellaria barbata* D.Don) 15g, *chì sháo* (the dried root of *Paeonia lactiflora* Pall.) 10g, *yán hú suǒ* (the dried tuber of *Corydalis yanhusuo* (Y.H.Chou & Chun C.Hsu) W.T.Wang ex Z.Y.Su & C.Y.Wu) 5g, *xiāng fù* (the dried rhizome of *Cyperus rotundus* L.) 15g, *wū yào* (the root tuber of *Lindera aggregata* (Sims) Kosterm.) 5g, *huáng qí* (the dried root of *Astragalus mongholicus* Bunge) 45g, *jiǎo gǔ lán* (the dried whole plant of *Gynostemma pentaphyllum* (Thunb.) Makin) 10g, *hóng huā* (the dried flower of *Carthamus tinctorius* L.) 10g, *gān cǎo* (the dried rhizome and root of *Glycyrrhiza glabra* L.) 6g, *é zhú* (the dried rhizoma of *Curcuma kwangsiensis* S.G.Lee & C.F.Liang) 10g, *sān léng* (the dried tuber of *Sparganium stoloniferum* (Buch.-Ham. ex Graebn.) Buch.-Ham. ex Juz.) 10g. If liver pain appeared, *sān qī* (the dried root and rhizome of *Panax notoginseng* (Burkill) F.H.Chen) 15g, *dì lóng* (the dried insect body of *Pheretima aspergillum* (E.Perrier)) 15g were added; if anorexia appeared, *mài yá* (the dried ripe fruit of *Hordeum vulgare* L.) 10g, *jiāo shān zhā* (the processing ripe fruit of *Crataegus pinnatifida* Bunge) 15g, *jiāo shén qǔ* (the dried yeast preparations of wheat bran, flour, ricebean powder (*Vigna umbellata* (Thunb.) Ohwi and Ohashi), and bitter apricot seed powder (*Prunus mandshurica* (Maxim.) Koehne) 15g, *jī nèi jīn* (the dried inner wall of the stomach of *Gallus gallus domesticus* Brisson) 10g were added; If abdominal distension appeared, *guì zhī* (the dried bark of *Cinnamomum cassia* (L.) J.Presl) 10g, *lái fú zǐ* (the dried ripe seed of *Raphanus raphanistrum subsp. sativus* (L.) Domin) 12g were added; if ascites appeared, *zhū líng* (a fungus, the dried sclerotium of *Polyporus umbellatus* (Pers.) Fries) 15g, *yì yǐ rén* (the dried seed of *Coix lacryma-jobi var. ma-yuen* (Rom.Caill.) Stapf) 40g, *zé xiè* (the dried tuber of Alisma *plantago-aquatica subsp. orientale* (Sam.) Sam.) 10g were added; If nausea and vomiting appeared, *shā rén* (the dried ripe fruit of *Wurfbainia villosa* (Lour.) Skornick. & A.D.Poulsen) 6g, *zhú rú* (the dried caulis of *Bambusa tuldoides* Munro) 10g were added; If fever appeared, *guì zhī* (the dried bark of *Cinnamomum cassia* (L.) J.Presl) 10g，*bò hé* (the dried aboveground part of *Mentha canadensis* L.) 20g, *chái hú* (the dried root of *Bupleurum chinense* DC.) 15g were added. |
| Ding R. F.2012^#^ | *huáng qí* (the dried root of *Astragalus mongholicus* Bunge) 40g, *tài zǐ shēn* (the dried toot tuber of *Pseudostellaria heterophylla* (Miq.) Pax) 10g, *tiān dōng* (the dried root tuber of *Asparagus cochinchinensis* (Lour.) Merr.) 10g, *bái zhú* (the dried rhizome of *Atractylodes macrocephala* Koidz.) 15g, *é zhú* (the dried rhizoma of *Curcuma kwangsiensis* S.G.Lee & C.F.Liang) 20g, *běi shā shēn* (the dried root of *Glehnia littoralis* (A.Gray) F.Schmidt ex Miq.) 15g, *hǔ zhàng* (the dried rhizome and root of *Reynoutria japonica* Houtt.) 30g, *zé xiè* (the dried tuber of Alisma *plantago-aquatica subsp. orientale* (Sam.) Sam.) 15g, *biē jiǎ* (the carapace of *Trionyx sinensis* Wiegmann) 10g, *zhū líng* (a fungus, the dried sclerotium of *Polyporus umbellatus* (Pers.) Fries) 15g, *dāng guī* (the dried root of *Angelica sinensis* (Oliv.) Diels) 10g, *mǔ lì* (the shell of *Ostrea gigas* Thunberg) 15g, *yán hú suǒ* (the dried tuber of *Corydalis yanhusuo* (Y.H.Chou & Chun C.Hsu) W.T.Wang ex Z.Y.Su & C.Y.Wu) 12g, *bái huā shé shé cǎo* (the dried whole plant of *Scleromitrion diffusum* (Willd.) R.J.Wang) 30g, *bái sháo* (the dried root of *Paeonia lactiflora* Pall.) 15g, *yì yǐ rén* (the dried seed of *Coix lacryma-jobi var. ma-yuen* (Rom.Caill.) Stapf) 30g, *gān cǎo* (the dried rhizome and root of *Glycyrrhiza glabra* L.) 6g. If nausea and vomiting appeared, *fǎ bàn xià* (the processing rhizome of *Pinellia ternata* (Thunb.) Makino), *zhú rú* (the dried caulis of *Bambusa tuldoides* Munro) were added; if jaundice appeared, *yīn chén* (the Aboveground Part of *Artemisia capillaris* Thunb.), *jīn qián cǎo* (the dried whole plant of *Lysimachia christinae* Hance) were added; if pain appeared, *xú zhǎng qīng* (the dried root and rhizome of Vincetoxicum mukdenense Kitag.), *pú huáng* (the dried pollen of *Typha angustifolia* L.), *wǔ* *líng zhī* (the dried fences of *Trogopterus xanthipes* Milne-Ed-wards) were added; if constipation appeared, *zhī mǔ* (the dried rhizome of *Anemarrhena asphodeloides* Bunge), *dà huáng* (the dried root and rhizome of Rheum palmatum L.) were added. |
| Zhang Q.2007^#^ | *huáng qí* (the dried root of *Astragalus mongholicus* Bunge), *dǎng shēn* (the dried root of *Codonopsis pilosula* (Franch.) Nannf.), *bái zhú* (the dried rhizome of *Atractylodes macrocephala* Koidz.), *fú líng* (a fungus, the dried sclerotium of Poria cocos (Schw.) Wolf), *jī xuè téng* (the dried stem of *Spatholobus suberectus* Dunn), *gǒu qǐ zǐ* (the dried ripe fruit of *Lycium barbarum* L.), *zhǒng jiē fēng* (the dried whole plant of *Sarcandra glabra* (Thunb.) Nakai), *nǚ zhēn zǐ* (the dried ripe fruit of *Ligustrum lucidum* W.T.Aiton), *qiàn cǎo* (the dried root and rhizome of *Rubia cordifolia* L.), *é zhú* (the dried rhizoma of *Curcuma kwangsiensis* S.G.Lee & C.F.Liang). |
| Zhang Z. Y.2017^#^ | *bàn zhī lián* (the dried whole plant of *Scutellaria barbata* D.Don) 15g, *bái huā shé shé cǎo* (the dried whole plant of *Scleromitrion diffusum* (Willd.) R.J.Wang) 30g, *huáng qí* (the dried root of *Astragalus mongholicus* Bunge) 30g, *é zhú* (the dried rhizoma of *Curcuma kwangsiensis* S.G.Lee & C.F.Liang) 15g, *sān léng* (the dried tuber of *Sparganium stoloniferum* (Buch.-Ham. ex Graebn.) Buch.-Ham. ex Juz.) 10g. |
| Li L.2017^#^ | *rén shēn* (the dried rhizome and root of *Panax ginseng* C.A.Mey.) 15g, *huáng qí* (the dried root of *Astragalus mongholicus* Bunge) 30g, *chǎo bái zhú* (the processing rhizome of *Atractylodes macrocephala* Koidz.) 15g, *fú líng* (a fungus, the dried sclerotium of Poria cocos (Schw.) Wolf) 15g, *chái hú* (the dried root of *Bupleurum chinense* DC.) 10g, *dāng guī* (the dried root of *Angelica sinensis* (Oliv.) Diels) 20g, *bái sháo* (the dried root of *Paeonia lactiflora* Pall.) 20g, *yù jīn* (the dried root tuber of *Curcuma aromatica* Salisb.) 15g, *yīn chén* (the Aboveground Part of *Artemisia capillaris* Thunb.) 15g, *bái huā shé shé cǎo* (the dried whole plant of *Scleromitrion diffusum* (Willd.) R.J.Wang) 15g, *bái tóu wēng* (the dried root of Pulsatilla chinensis (Bunge) Regel) 15g, *ròu guì* (the dried bark of *Neolitsea cassia* (L.) Kosterm.) 3g, *zhì gān cǎo* (the processing rhizome and root of *Glycyrrhiza glabra* L.) 5g, *sān qī* (the dried root and rhizome of *Panax notoginseng* (Burkill) F.H.Chen) 3g, *é zhú* (the dried rhizoma of *Curcuma kwangsiensis* S.G.Lee & C.F.Liang) 15g. |

^△^Represent CMFs adopt the therapeutic principles of PSSQ; ^#^ Represent CMFs adopt the therapeutic principles of PCSQEP

## Supplementary table 4

Adverse Events of intervention, including a comparison between PSSQ and PCSQEP in the subgroup.

| ***Item*** | ***First author and year*** | ***Group*** | ***Trials*** | ***I^2^*** | ***RR*** | ***95%CI*** | ***p-value*** |
| --- | --- | --- | --- | --- | --- | --- | --- |
| **Entire adverse events** | Li L.2017, Du H. P.2018, Jiang R. R.2020 | PSSQ | 0 | \ | \ | \ | \ |
|  |  | PCSQEP | 3 | 76% | 0.87 | 0.47-1.59 | 0.64 |
|  |  | Overall | 3 | 76% | 0.87 | 0.47-1.59 | 0.64 |
| **Nausea and vomit** | Shen L. N.2020, Cheng M. F.2015, Huang J. Y.2009, Du H. P.2018, Li L.2017, Jiang R. R.2020, Wang Q. M.2016 | PSSQ | 2 | 0% | 0.68 | 0.48-0.97 | 0.03 |
|  |  | PCSQEP | 5 | 12% | 0.73 | 0.55-0.97 | 0.03 |
|  |  | Overall | 7 | 0% | 0.71 | 0.57-0.89 | 0.02 |
| **Inappetence** | Shen L. N.2020, Du H. P.2018, Li L.2017 | PSSQ | 1 | \ | 0.65 | 0.43-0.98 | 0.04 |
|  |  | PCSQEP | 2 | 0% | 0.66 | 0.40-1.11 | 0.12 |
|  |  | Overall | 3 | 0% | 0.66 | 0.47-0.91 | 0.01 |
| **Digestive symptoms** | Cheng M. F.2015, Ding R. F.2012, Huang J. Y.2009, Wang Q. M.2016, Jiang R. R.2020 | PSSQ | 1 | \ | 0.77 | 0.48-1.23 | 0.28 |
|  |  | PCSQEP | 4 | 10% | 0.66 | 0.52-0.82 | 0.0003 |
|  |  | Overall | 5 | 0% | 0.68 | 0.55-0.84 | 0.0002 |
| **Digestive symptoms Ⅰ** | Wang A. M.2020, Ye W. D.2015, Ding R. F.2012 | PSSQ | 2 | 0% | 2.36 | 1.65-3.38 | ＜0.00001 |
|  |  | PCSQEP | 1 | \ | 0.51 | 0.33-0.78 | 0.002 |
|  |  | Overall | 3 | 93% | 1.41 | 0.49-4.07 | 0.53 |
| **Digestive symptoms Ⅱ Ⅲ** | Wang A. M.2020, Ye W. D.2015, Ding R. F.2012 | PSSQ | 2 | 0% | 0.28 | 0.16-0.48 | ＜0.00001 |
|  |  | PCSQEP | 1 | \ | 1.14 | 0.34-3.84 | 0.84 |
|  |  | Overall | 3 | 54% | 0.39 | 0.18-0.83 | 0.01 |
| **Fever** | Shen L. N.2020, Li L.2017, Jiang R. R.2020 | PSSQ | 1 | \ | 0.42 | 0.17-1.04 | 0.06 |
|  |  | PCSQEP | 2 | 0% | 0.88 | 0.65-1.18 | 0.39 |
|  |  | Overall | 3 | 29% | 0.77 | 0.58-1.03 | 0.08 |
| **Fever Ⅰ** | Wang A. M.2020, Ye W. D.2015 | PSSQ | 2 | 0% | 1.72 | 1.28-2.32 | 0.0004 |
|  |  | PCSQEP | 0 | \ | \ | \ | \ |
|  |  | Overall | 2 | 0% | 1.72 | 1.28-2.32 | 0.0004 |
| **Fever Ⅱ Ⅲ** | Wang A. M.2020, Ye W. D.2015 | PSSQ | 2 | 0% | 0.40 | 0.24-0.67 | 0.0005 |
|  |  | PCSQEP | 0 | \ | \ | \ | \ |
|  |  | Overall | 2 | 0% | 0.40 | 0.24-0.67 | 0.0005 |
| **Liver damage** | Li Y. H.2011, Jiang R. R.2020 | PSSQ | 1 | \ | 0.87 | 0.46-1.66 | 0.68 |
|  |  | PCSQEP | 1 | \ | 0.92 | 0.47-1.80 | 0.81 |
|  |  | Overall | 2 | 0% | 0.90 | 0.57-1.43 | 0.65 |
| **Liver damage Ⅰ** | Wang A. M.2020, Ye W. D.2015 | PSSQ | 2 | 0% | 1.88 | 1.34-2.64 | 0.0003 |
|  |  | PCSQEP | 0 | \ | \ | \ | \ |
|  |  | Overall | 2 | 0% | 1.88 | 1.34-2.64 | 0.0003 |
| **Liver damage Ⅱ Ⅲ** | Wang A. M.2020, Ye W. D.2015 | PSSQ | 2 | 0% | 0.44 | 0.28-0.68 | 0.0003 |
|  |  | PCSQEP | 0 | \ | \ | \ | \ |
|  |  | Overall | 2 | 0% | 0.44 | 0.28-0.68 | 0.0003 |
| **Bone marrow suppression** | Li Y. H.2011, Ding R. F.2012, Li L.2017 | PSSQ | 1 | \ | 0.61 | 0.39-0.93 | 0.02 |
|  |  | PCSQEP | 2 | 52% | 0.56 | 0.32-0.98 | 0.04 |
|  |  | Overall | 3 | 8% | 0.58 | 0.43-0.78 | 0.0004 |
| **WBC decline** | Chi H. C.2010, Ding R. F.2012, Huang J. Y.2009, Zhang Q.2007, Li L.2017 | PSSQ | 1 | \ | 0.93 | 0.76-1.16 | 0.53 |
|  |  | PCSQEP | 4 | 85% | 0.71 | 0.44-1.13 | 0.14 |
|  |  | Overall | 5 | 79% | 0.79 | 0.58-1.07 | 0.12 |
| **WBC decline Ⅰ** | Chi H. C.2010, Ye W. D.2015, Ding R. F.2012, Zhang Q.2007 | PSSQ | 2 | 0% | 1.88 | 1.26-2.78 | 0.002 |
|  |  | PCSQEP | 2 | 0% | 1.25 | 0.82-1.91 | 0.29 |
|  |  | Overall | 4 | 0% | 1.55 | 1.16-2.07 | 0.003 |
| **WBC decline Ⅱ Ⅲ Ⅳ** | Chi H. C.2010, Ye W. D.2015, Ding R. F.2012, Zhang Q.2007, Li L.2017 | PSSQ | 2 | 0% | 0.57 | 0.41-0.79 | 0.0008 |
|  |  | PCSQEP | 3 | 26% | 0.83 | 0.59-1.18 | 0.30 |
|  |  | Overall | 5 | 25% | 0.68 | 0.54-0.87 | 0.002 |
| **Blood platelet reduction** | Chi H. C.2010, Ding R. F.2012, Zhang Q.2007 | PSSQ | 1 | \ | 0.89 | 0.67-1.20 | 0.46 |
|  |  | PCSQEP | 2 | 0% | 0.92 | 0.76-1.12 | 0.40 |
|  |  | Overall | 3 | 0% | 0.91 | 0.77-1.07 | 0.26 |
| **Blood platelet reduction Ⅰ** | Chi H. C.2010, Ding R. F.2012, Zhang Q.2007 | PSSQ | 1 | \ | 0.77 | 0.37-1.62 | 0.49 |
|  |  | PCSQEP | 2 | 0% | 0.88 | 0.52-1.48 | 0.62 |
|  |  | Overall | 3 | 0% | 0.84 | 0.54-1.28 | 0.41 |
| **Blood platelet reduction Ⅱ Ⅲ** | Chi H. C.2010, Ding R. F.2012, Zhang Q.2007 | PSSQ | 1 | \ | 0.71 | 0.34-1.48 | 0.37 |
|  |  | PCSQEP | 2 | 0% | 0.88 | 0.54-1.43 | 0.60 |
|  |  | Overall | 3 | 0% | 0.82 | 0.54-1.23 | 0.33 |
| **Hemoglobin reduction** | Chi H. C.2010, Ding R. F.2012, Zhang Q.2007 | PSSQ | 1 | \ | 0.95 | 0.73-1, 24 | 0.70 |
|  |  | PCSQEP | 2 | 0% | 0.98 | 0.76-1.26 | 0.88 |
|  |  | Overall | 3 | 0% | 0.97 | 0.81-1.16 | 0.72 |
| **Hemoglobin reduction Ⅰ** | Chi H. C.2010, Ding R. F.2012, Zhang Q.2007 | PSSQ | 1 | \ | 1.00 | 0.59-1.69 | 1 |
|  |  | PCSQEP | 2 | 0% | 0.89 | 0.58-1.36 | 0.60 |
|  |  | Overall | 3 | 0% | 0.93 | 0.67-1.30 | 0.68 |
| **Hemoglobin reduction Ⅱ Ⅲ** | Chi H. C.2010, Ding R. F.2012, Zhang Q.2007 | PSSQ | 1 | \ | 1.82 | 0.50-6.64 | 0.37 |
|  |  | PCSQEP | 2 | 0% | 0.94 | 0.65-1.37 | 0.75 |
|  |  | Overall | 3 | 0% | 1.01 | 0.70-1.44 | 0.97 |
| **Neurotoxicity** | Ding R. F.2012, Cheng M. F.2015, Wang Q. M.2016 | PSSQ | 1 | \ | 0.14 | 0.01-2.68 | 0.19 |
|  |  | PCSQEP | 2 | 58% | 0.80 | 0.30-2.12 | 0.65 |
|  |  | Overall | 3 | 50% | 0.66 | 0.25-1.80 | 0.42 |
