# Supplementary material for: The Therapeutic Principle of Combined Strengthening Qi and Eliminating Pathogens in Treating Middle-Advanced Primary Liver Cancer: A Systematic Review and Meta-Analysis
Source: Front Pharmacol. 2021 Oct 27;12:714287. doi: 10.3389/fphar.2021.714287 (PMC8578139; doi:10.3389/fphar.2021.714287)
Supplement: Supplementary file 10 [file DataSheet1.DOCX]

**Supplementary Data**

**PubMed Search formation**

(((((randomized controlled trial[pt] OR controlled clinical trial[pt] OR randomized[tiab] OR placebo[tiab] OR clinical trials as topic[mesh:noexp] OR randomly[tiab] OR trial[ti]) NOT (animals [mh] NOT humans [mh])) NOT (meta[Title/Abstract])) AND ((TACE[Title/Abstract]) OR (chemoembolization*[Title/Abstract]))) AND ((((((drugs, chinese herbal[MeSH Terms]) OR (MEDICINE, CHINESE TRADITIONAL[MeSH Terms])) OR (((((((Herb*[Title/Abstract]) OR (drug*[Title/Abstract])) OR (plant*[Title/Abstract])) OR (extract*[Title/Abstract])) OR (Medicine[Title/Abstract])) OR (formula[Title/Abstract])) AND (Chinese[Title/Abstract]))) OR ((DECOCTION*[Title/Abstract]) OR (herba*[Title/Abstract]))) OR (Jianpi[Title/Abstract] OR ((Invigorat*[Title/Abstract] OR Strength*[Title/Abstract] OR Reinforc*[Title/Abstract] OR activ*[Title/Abstract] OR Tonify*[Title/Abstract]) AND Spleen[Title/Abstract]))) OR (qi[MeSH Terms] OR qi[Title/Abstract] OR (Vital Energy[Title/Abstract])))) AND ((((((((((Neoplasms[MeSH Terms]) OR (Carcinoma[MeSH Terms])) OR (Neoplasia*[Title/Abstract])) OR (Tumor*[Title/Abstract])) OR (Neoplasm*[Title/Abstract])) OR (Cancer*[Title/Abstract])) OR (Carcinoma*[Title/Abstract])) OR (Epithelioma*[Title/Abstract])) AND ((((((((((Liver[MeSH Terms]) OR (LIVER*[Title/Abstract])) OR (Hepatic[Title/Abstract])) OR (Hepatocellular[Title/Abstract])) OR (Liver*[tiab] AND Cell[tiab])) OR (Cholangiocellular[Title/Abstract])) OR (bile tract[Title/Abstract])) OR (bile duct[Title/Abstract])) OR (gall duct[Title/Abstract])) OR (biliary[Title/Abstract]))) OR ((((((HCC[Title/Abstract]) OR (Hepatoma*[Title/Abstract])) OR (Cholangiocarcinoma[MeSH Terms])) OR (Cholangiocarcinoma*[Title/Abstract])) OR (ICC[Title/Abstract])) OR (PLC[Title/Abstract])))

**EMBASE Search formation**

#14 #5 AND #11 AND #12 AND #13

#13 'crossover procedure':de OR 'double-blind procedure':de OR 'randomized controlled trial':de OR 'single-blind procedure':de OR random*:de,ab,ti OR factorial*:de,ab,ti OR crossover*:de,ab,ti OR ((cross NEXT/1 over*):de,ab,ti) OR placebo*:de,ab,ti OR ((doubl* NEAR/1 blind*):de,ab,ti) OR ((singl* NEAR/1 blind*):de,ab,ti) OR assign*:de,ab,ti OR allocat*:de,ab,ti OR volunteer*:de,ab,ti

#12 'chemoembolization'/exp OR 'chemoembolization*':ab,ti OR 'tace':ab,ti

#11 #6 OR #7 OR #8 OR #9 OR #10

#10 'qi'/exp OR 'qi':ab,ti OR 'vital energy':ab,ti

#9 'jianpi':ab,ti OR ((invigorat*:ab,ti OR strength*:ab,ti OR reinforc*:ab,ti OR activ*:ab,ti OR tonify*:ab,ti) AND spleen:ab,ti)

#8 'decoction*':ab,ti OR 'herba*':ab,ti

#7 ('herb*':ab,ti OR 'drug*':ab,ti OR 'plant*':ab,ti OR 'extract*':ab,ti OR 'medicine':ab,ti OR 'formula':ab,ti) AND 'chinese':ab,ti

#6 'herbaceous agent'/exp OR 'chinese medicine'/exp

#5 #4 OR #3

#4 #1 AND #2

#3 'liver tumor'/exp OR 'hepatoma*':ab,ti OR 'hcc':ab,ti OR 'bile duct carcinoma'/exp OR 'cholangiocarcinoma*':ab,ti OR 'icc':ab,ti OR 'plc':ab,ti

#2 'neoplasm'/exp OR 'carcinoma'/exp OR 'neoplasia*':ab,ti OR 'tumor*':ab,ti OR 'neoplasm*':ab,ti OR 'cancer*':ab,ti OR 'carcinoma*':ab,ti OR 'epithelioma*':ab,ti

#1 'liver'/exp OR 'liver*':ab,ti OR 'hepatic':ab,ti OR 'hepatocellular':ab,ti OR ('liver*':ab,ti AND 'cell':ab,ti) OR 'bile tract':ab,ti OR 'bile duct':ab,ti OR 'gall duct':ab,ti OR 'biliary':ab,ti OR 'cholangiocellular':ab,ti

**Web of Science search formation**

#13 #12 AND #11 AND #10 AND #5

#12 TS=clinical trial* OR TS=research design OR TS=comparative stud* OR TS=evaluation stud* OR TS=controlled trial* OR TS=follow-up stud* OR TS=prospective stud* OR TS=random* OR TS=placebo* OR TS=(single blind*) OR TS=(double blind*)

#11 TS=('chemoembolization* OR TACE)

#10 #6 OR #7 OR #8 OR #9 OR #10

#9 TS=(qi OR “vital energy”)

#8 TS=(((Invigorat* OR Strength* OR Reinforc* OR activ* OR Tonify*) AND spleen) OR JIANPI)

#7 TS=(Decoction* OR herba*)

#6 TS=((Herb* OR drug* OR plant* OR extract* OR Medicine OR formula) AND Chinese)

#5 #4 OR #3

#4 #1 AND #2

#3 TS=(HCC OR Hepatoma* OR Cholangiocarcinoma* OR ICC OR PLC

#2 TS=(Neoplasia* OR Tumor* OR Neoplasm* OR Cancer* OR Carcinoma* OR Epithelioma*)

#1 TS=(LIVER* OR Hepatic OR Hepatocellular OR Liver* SAME Cell) OR Cholangiocellular OR “bile tract” OR “bile duct” OR “gall duct” OR “biliary”)

**Cochrane search formation**

#1 MeSH descriptor: [Liver] explode all trees 3192

#2 (LIVER*):ti,ab,kw (Word variations have been searched) 50737

#3 (Hepatocellular):ti,ab,kw (Word variations have been searched) 5179

#4 (Hepatic):ti,ab,kw (Word variations have been searched) 36228

#5 (Liver*):ti,ab,kw (Word variations have been searched) 50737

#6 (Cell):ti,ab,kw (Word variations have been searched) 133073

#7 (Cholangiocellular):ti,ab,kw (Word variations have been searched) 19

#8 (bile tract):ti,ab,kw (Word variations have been searched) 904

#9 (bile duct):ti,ab,kw (Word variations have been searched) 2984

#10 (gall duct):ti,ab,kw (Word variations have been searched) 87

#11 (biliary):ti,ab,kw (Word variations have been searched) 5683

#12 #5 AND #6 9289

#13 #1 OR #2 OR #3 OR #4 OR #7 OR #8 OR #9 OR #10 OR #11 OR #12 76147

#14 MeSH descriptor: [Neoplasms] explode all trees 79635

#15 MeSH descriptor: [Carcinoma] explode all trees 13534

#16 (Neoplasia*):ti,ab,kw (Word variations have been searched) 2746

#17 (Tumor*):ti,ab,kw (Word variations have been searched) 64913

#18 (Neoplasm*):ti,ab,kw (Word variations have been searched) 79643

#19 (Cancer*):ti,ab,kw (Word variations have been searched) 161872

#20 (Carcinoma*):ti,ab,kw (Word variations have been searched) 40936

#21 (Epithelioma*):ti,ab,kw (Word variations have been searched) 16

#22 #14 OR #15 OR #16 OR #17 OR #18 OR #19 OR #20 OR #21 219004

#23 (HCC):ti,ab,kw (Word variations have been searched) 3282

#24 (Hepatoma*):ti,ab,kw (Word variations have been searched) 132

#25 MeSH descriptor: [Cholangiocarcinoma] explode all trees 216

#26 (Cholangiocarcinoma*):ti,ab,kw (Word variations have been searched) 687

#27 (ICC):ti,ab,kw 1749

#28 (PLC):ti,ab,kw 597

#29 #23 OR #24 OR #25 OR #26 OR #27 OR 28 197364

#30 #13 AND #22 19976

#31 #30 OR #29 210979

#32 MeSH descriptor: [Drugs, Chinese Herbal] explode all trees 3576

#33 MeSH descriptor: [Medicine, Chinese Traditional] explode all trees 1179

#34 #32 OR #33 4209

#35 (Herb*):ti,ab,kw (Word variations have been searched) 11375

#36 (drug*):ti,ab,kw (Word variations have been searched) 614910

#37 (plant*):ti,ab,kw (Word variations have been searched) 18308

#38 (extract*):ti,ab,kw (Word variations have been searched) 41748

#39 (Medicine):ti,ab,kw (Word variations have been searched) 58388

#40 (formula):ti,ab,kw (Word variations have been searched) 11925

#41 (Chinese):ti,ab,kw (Word variations have been searched) 27506

#42 #35 OR #36 OR #37 OR #38 OR #39 OR #40 685834

#43 #42 AND #41 19697

#44 (DECOCTION*):ti,ab,kw (Word variations have been searched) 3516

#45 (herba*):ti,ab,kw (Word variations have been searched) 9621

#46 #44 OR #45 12408

#47 #34 OR #43 OR #46 26255

#48 (Jianpi):ti,ab,kw (Word variations have been searched) 234

#49 (Invigorat*):ti,ab,kw (Word variations have been searched) 281

#50 (Strength*):ti,ab,kw (Word variations have been searched) 44724

#51 (Reinforc*):ti,ab,kw (Word variations have been searched) 8524

#52 (activ*):ti,ab,kw (Word variations have been searched) 274354

#53 (Tonify*):ti,ab,kw (Word variations have been searched) 254

#54 (Spleen):ti,ab,kw (Word variations have been searched) 2584

#55 #49 OR #50 OR #51 OR #52 OR #53 310364

#56 #55 AND #54 723

#57 #48 OR #56 934

#58 MeSH descriptor: [Qi] explode all trees 245

#59 (qi):ti,ab,kw (Word variations have been searched) 2733

#60 (Vital Energy):ti,ab,kw (Word variations have been searched) 590

#61 #58 OR #59 OR #60 3397

#62 #47 OR #57 OR #61 28858

#63 (TACE):ti,ab,kw (Word variations have been searched) 1119

#64 (chemoembolization*):ti,ab,kw (Word variations have been searched) 1250

#65 #63 OR #64 1542

#66 #31 AND #62 AND #65 66

**CNKI search formation**

TKA=('肝癌'+'肝恶性肿瘤'+'原发性肝细胞癌'+'肝脏恶性肿瘤'+'肝肿瘤'+'肝细胞癌'+'肝内胆管癌'+'PLC'+'HCC'+'ICC') AND FT=('介入治疗'+'经动脉化疗栓塞术'+'肝动脉插管化疗栓塞术'+'肝动脉栓塞化疗'+'经肝动脉栓塞化疗'+'肝动脉插管化疗栓塞'+'肝动脉栓塞灌注化疗'+'经导管动脉化疗栓塞术'+'经肝动脉栓塞化疗术'+'肝动脉化疗栓塞术'+'经皮肝动脉化疗栓塞术'+'肝动脉栓塞化疗术'+'经肝动脉化疗栓塞'+'经导管动脉栓塞化疗'+'经肝动脉化疗栓塞术'+'介入性治疗'+'动脉化疗栓塞术'+'经导管肝动脉化疗栓塞术'+'经导管肝动脉化疗栓塞'+'经导管动脉化学栓塞'+'肝动脉化疗栓塞'+'经皮肝动脉化疗栓塞'+'肝动脉介入化疗'+'插管化疗栓塞'+'肝动脉灌注化疗栓塞'+'肝动脉灌注化疗栓塞术'+'经导管动脉化疗栓塞'+'介入化疗栓塞'+'介入疗法'+'经动脉栓塞化疗'+'经动脉化疗栓塞'+'TACE') AND TKA=('临床观察'+'clinical observation'+'疗效'+'体会'+'应用'+'评价'+'临床效果'+'临床研究'+'随机对照试验'+'randomized clinical trials'+'Randomized controlled clinical trial'+'randomized controlled trial'+'randomized controlled trials'+'randomized experiment'+'rct'+'随机对照实验'+'随机对照研究'+'随机对照') NOT TKA=('meta'+'荟萃分析'+'元分析'+'系统评价') AND (TI=('汤'+'方'+'丸'+'法'+'复方'+'合剂') OR FT=('中药'+'中医'+'中西')) NOT TI='研究进展'

**Wanfang database search formation**

主题:("肝癌" or "肝恶性肿瘤" or "原发性肝细胞癌" or "肝脏恶性肿瘤" or "肝肿瘤" or "肝细胞癌" or "肝内胆管癌" or "PLC" or "HCC" or "ICC") AND 全部:("介入治疗" or "经动脉化疗栓塞术" or “肝动脉插管化疗栓塞术” or “肝动脉栓塞化疗” or “经肝动脉栓塞化疗” or “肝动脉插管化疗栓塞” or “肝动脉栓塞灌注化疗” or “经导管动脉化疗栓塞术” or “经肝动脉栓塞化疗术” or “肝动脉化疗栓塞术” or “经皮肝动脉化疗栓塞术” or “肝动脉栓塞化疗术” or “经肝动脉化疗栓塞” or “经导管动脉栓塞化疗” or “经肝动脉化疗栓塞术” or “介入性治疗” or “动脉化疗栓塞术” or “经导管肝动脉化疗栓塞术” or “经导管肝动脉化疗栓塞” or “经导管动脉化学栓塞” or “肝动脉化疗栓塞” or “经皮肝动脉化疗栓塞” or “肝动脉介入化疗” or “插管化疗栓塞” or “肝动脉灌注化疗栓塞” or “肝动脉灌注化疗栓塞术” or “经导管动脉化疗栓塞” or “介入化疗栓塞” or “介入疗法” or "经动脉栓塞化疗" or "经动脉化疗栓塞" or "TACE") AND 主题:(“临床观察” or ”clinical observation” or ”疗效” or ”体会” or ”应用” or ”评价” or ”临床效果” or ”临床研究” or ”随机对照试验” or ”randomized clinical trials” or ”Randomized controlled clinical trial” or ”randomized controlled trial” or ”randomized controlled trials” or ”randomized experiment” or ”rct” or ”随机对照实验” or ”随机对照研究” or ”随机对照”) NOT 主题:(“meta” or ”荟萃分析” or ”元分析” or ”系统评价”) AND (题名:(“汤” or ”方” or ”丸” or ”法” or ”复方” or ”合剂”) OR 全部：(“中药” or ”中医” or ”中西”))

**CBM search formation**

("肝癌”[常用字段:智能] OR ”肝恶性肿瘤”[常用字段:智能] OR ”原发性肝细胞癌”[常用字段:智能] OR ”肝脏恶性肿瘤”[常用字段:智能] OR ”肝肿瘤”[常用字段:智能] OR ”肝细胞癌”[常用字段:智能] OR ”肝内胆管癌”[常用字段:智能] OR ”PLC”[常用字段:智能] OR ”HCC”[常用字段:智能] OR ”ICC” [常用字段:智能]) AND (“介入治疗”[全部字段:智能] OR ”经动脉化疗栓塞术”[全部字段:智能] OR ”肝动脉插管化疗栓塞术”[全部字段:智能] OR ”肝动脉栓塞化疗”[全部字段:智能] OR ”经肝动脉栓塞化疗”[全部字段:智能] OR ”肝动脉插管化疗栓塞”[全部字段:智能] OR ”肝动脉栓塞灌注化疗”[全部字段:智能] OR ”经导管动脉化疗栓塞术”[全部字段:智能] OR ”经肝动脉栓塞化疗术”[全部字段:智能] OR ”肝动脉化疗栓塞术”[全部字段:智能] OR ”经皮肝动脉化疗栓塞术”[全部字段:智能] OR ”肝动脉栓塞化疗术”[全部字段:智能] OR ”经肝动脉化疗栓塞”[全部字段:智能] OR ”经导管动脉栓塞化疗”[全部字段:智能] OR ”经肝动脉化疗栓塞术”[全部字段:智能] OR ”介入性治疗”[全部字段:智能] OR ”动脉化疗栓塞术”[全部字段:智能] OR ”经导管肝动脉化疗栓塞术”[全部字段:智能] OR ”经导管肝动脉化疗栓塞”[全部字段:智能] OR ”经导管动脉化学栓塞”[全部字段:智能] OR ”肝动脉化疗栓塞”[全部字段:智能] OR ”经皮肝动脉化疗栓塞”[全部字段:智能] OR ”肝动脉介入化疗”[全部字段:智能] OR ”插管化疗栓塞”[全部字段:智能] OR ”肝动脉灌注化疗栓塞”[全部字段:智能] OR ”肝动脉灌注化疗栓塞术”[全部字段:智能] OR ”经导管动脉化疗栓塞”[全部字段:智能] OR ”介入化疗栓塞”[全部字段:智能] OR ”介入疗法”[全部字段:智能] OR ”经动脉栓塞化疗”[全部字段:智能] OR ”经动脉化疗栓塞”[全部字段:智能] OR ”TACE” [全部字段:智能]) AND ((“汤”[常用字段:智能] OR ”方”[常用字段:智能] OR ”丸”[常用字段:智能] OR ”法”[常用字段:智能] OR ”复方”[常用字段:智能] OR ”合剂”) OR (“中药”[全部字段:智能] OR ”中医”[全部字段:智能] OR ”中西” [全部字段:智能]))

限定条件： 临床试验; 随机对照试验; 多中心研究; 人类;

**VIP search formation**

(M=(肝癌+肝恶性肿瘤+原发性肝细胞癌+肝脏恶性肿瘤+肝肿瘤+肝细胞癌+肝内胆管癌+PLC+HCC+ICC)+R=(肝癌+肝恶性肿瘤+原发性肝细胞癌+肝脏恶性肿瘤+肝肿瘤+肝细胞癌+肝内胆管癌+PLC+HCC+ICC))*U=(介入治疗+动脉化疗栓塞+肝动脉插管化疗栓塞术+动脉栓塞化疗+肝动脉插管化疗栓塞+肝动脉栓塞灌注化疗+介入性治疗+经导管动脉化学栓塞+插管化疗栓塞+肝动脉灌注化疗栓塞术+介入化疗栓塞+介入疗法+TACE)*(M=(临床观察+clinical observation+疗效+体会+应用+评价+临床效果+临床研究+随机对照试验+randomized clinical trials+Randomized controlled clinical trial+randomized controlled trial+randomized controlled trials+randomized experiment+rct+随机对照实验+随机对照研究+随机对照)+R=(临床观察+clinical observation+疗效+体会+应用+评价+临床效果+临床研究+随机对照试验+randomized clinical trials+Randomized controlled clinical trial+randomized controlled trial+randomized controlled trials+randomized experiment+rct+随机对照实验+随机对照研究+随机对照))*(M=(汤+方+丸+法+复方+合剂)+R=(汤+方+丸+法+复方+合剂)+U=(中药+中医+中西))-M=(meta+荟萃分析+元分析+系统评价+研究进展)
